# Supplementary material for: Innate Lymphoid Cell Activation and Sustained Depletion in Blood and Tissue of Children Infected with HIV from Birth Despite Antiretroviral Therapy
Source: Cell Rep. 2020 Sep 15;32(11):108153. doi: 10.1016/j.celrep.2020.108153 (PMC7495043; doi:10.1016/j.celrep.2020.108153)
Supplement: Document S1. Figures S1–S4 [file mmc1.pdf]

Cell Reports, Volume *32*

Supplemental Information

Innate Lymphoid Cell Activation and Sustained Depletion in Blood and Tissue of Children Infected with HIV from Birth Despite Antiretroviral Therapy

Alveera Singh, Samuel W. Kazer, Julia Roider, Kami C. Krista, Jane Millar, Osaretin E. Asowata, Abigail Ngoepe, Duran Ramsuran, Rabiah Fardoos, Amanda Ardain, Maximilian Muenchhoff, Warren Kuhn, Farina Karim, Thumbi Ndung'u, Alex K. Shalek, Philip Goulder, Alasdair Leslie, and Henrik N. Kløverpris

Supplementary Material

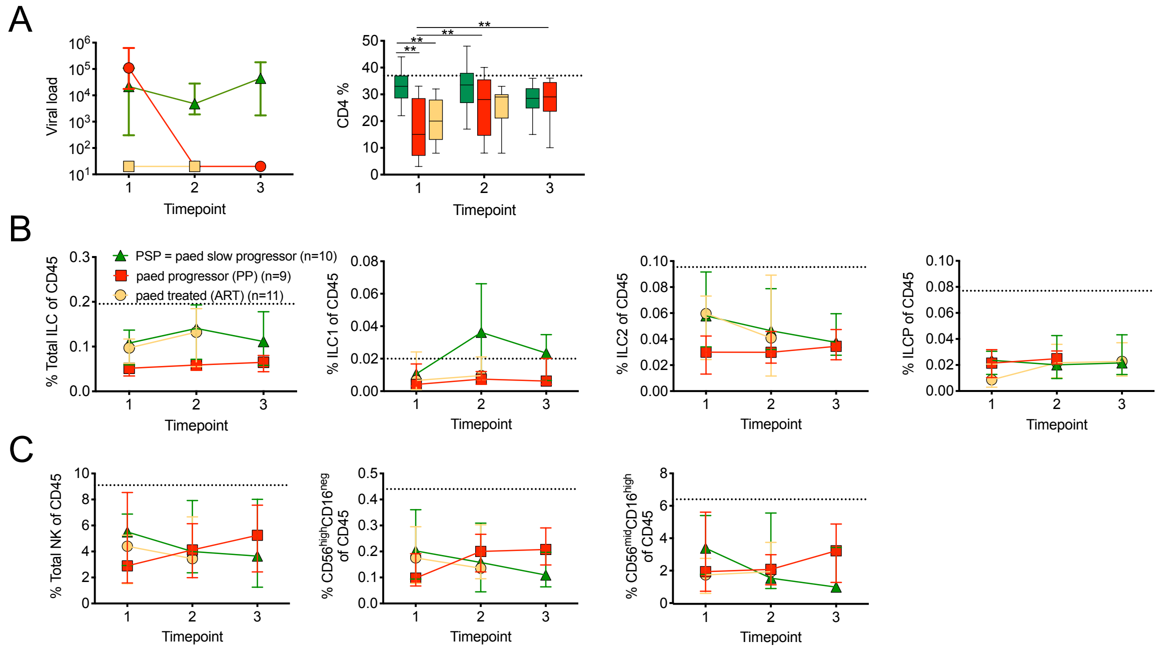

Figure S1. Related to Figure 3. Sustained depletion of all ILC subsets during treated paediatric HIV infection.. (**A**) Plasma viral load (left) and CD4 percentage (right) of 3 paediatric HIV groups: 1. Paediatric slow progressors (PSP, green, n=12), 2. Paediatric progressors (PP, red, n=11) and 3. Paediatric treated (ART, yellow, n=11). (**B**) Same longitudinal sampling as in (A) but showing longitudinal sampling of all blood (total ILC: top row, right; blue), helper (ILC1: middle row, left; yellow; ILC2: middle row, center; red), precursor (ILCP: middle row, right, light blue) and (**C**) cytotoxic subsets (NK cells: bottom row). *P*-values by Kruskall-Wallis multiple comparison.

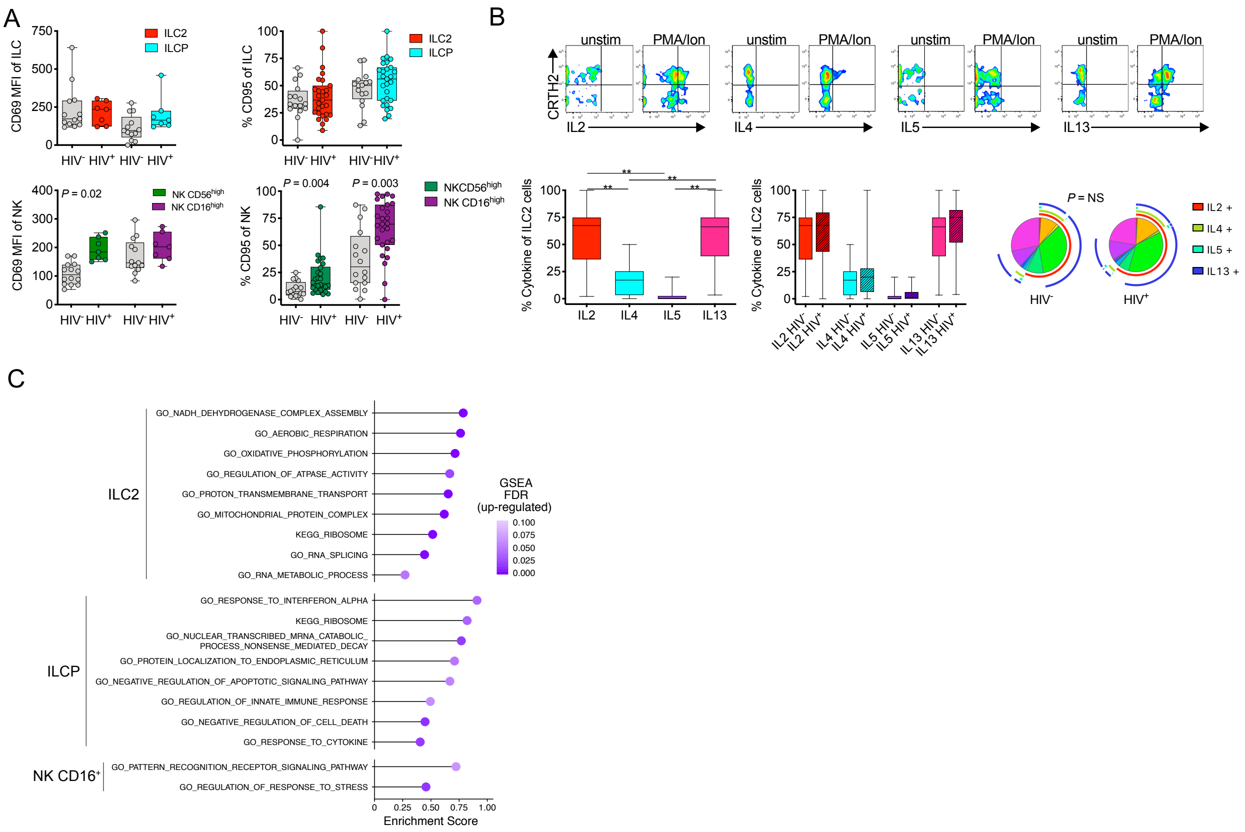

Figure S2. Related to Figure 4. Circulating NK cells, but not helper ILCs, upregulate activation markers in HIV-infected children with ILC2s and ILCPs upregulating genes enriched for metabolic GO and KEGG terms in paediatric HIV infection. (**A**) CD69 median fluorescence intensity (MFI) of CD69 and CD95 gated on ILC2 and ILCP (top) and NK CD56^{high} and NK CD16^{high} subsets (bottom) in blood. (**B**) Intracellular cytokine staining of ILC2 cells after 4 hrs PMA/Ion stimulation. (**C**) GSEA enrichment score and significance plotted of representative hits for DEGs (FDR corrected q<0.1; see Table S3 for genes) for ILC2s, ILC3s, and NK CD16^{+}cells between HIV infected children and healthy controls. See Table S4 for full GSEA results; performed using the GO and KEGG terms available on MSigDB (v7.0).

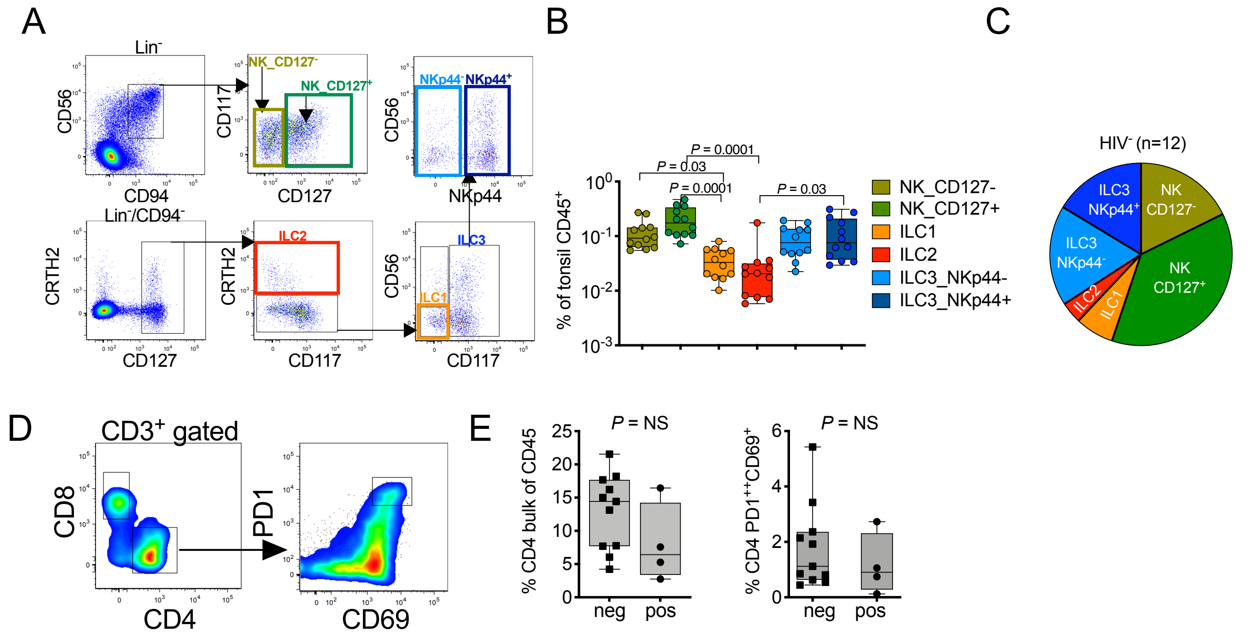

Figure S3. Related to Figure 5. Gating strategy to identify frequencies of helper ILCs and NK cells in paediatric tonsils and with unchanged frequencies of CD4⁺ T cells in HIV infected paediatric tonsils. (**A**) Gating strategy to identify 4 helper and 2 cytotoxic ILC populations in paediatric tonsil lymphocytes from lineage negative (Lin⁻) cells (CD3, CD4, CD19, CD14, TCRαβ, TCRγδ, CD11c). (**B**) Frequencies of each of these 6 innate lymphocyte populations expressed as % of CD45 cells with horizontal bar indicating median values of 12 HIV negative paediatric tonsils. (**C**) Relative frequencies for each of the 6 populations gated as in A. (**D**) Gating strategy to identify frequencies of CD4⁺ bulk T cells and T-follicular helper cells in paediatric tonsils. (**E**) Frequencies expressed as % of CD45 cells with horizontal bar indicating median values of comparison of 12 HIV negative paediatric tonsils against 4 HIV infected paediatric tonsils.

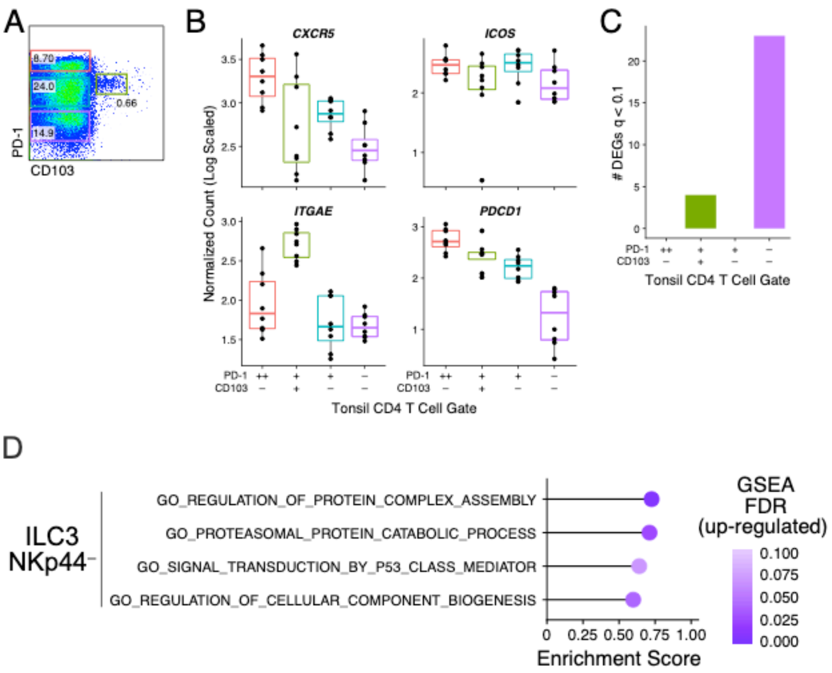

Figure S4. Related to Figure 6. CD4$^+$ T cell subsets from tonsils of HIV infected and HIV uninfected children and ILC3 NKp44$^-$ cells, but not ILC3 NKp44$^+$ or NK CD127$^-$ cells, upregulating genes associated with protein production and activation. (**A**) Sort gates used on CD4$^+$CD8$^-$ cells to isolate (1) PD-1^{++}CD103$^-$ [red]; (2) PD-1$^+$CD103$^+$ [green]; (3) PD-1$^+$CD103$^-$ [blue]; and (4) PD-1$^-$CD103$^-$ [purple] subsets. (**B**) Gene expression of *CXCR5, ICOS, ITGAE* (CD103), and *PDCD1* (PD-1) across subsets and participants. Each dot represents the average of 1-3 biological replicates of the subset for a given participant. (**C**) Number of differentially expressed genes for each tonsil CD4$^+$ T cell subset between HIV infected (n=3; 1 male + 2 female) and HIV uninfected (n=5; 4 male + 1 female) children. FDR q calculated using DESeq2. **(D)** GSEA enrichment score and significance plotted of representative hits for DEGs (FDR corrected q<0.1; see Table S7 for genes) for ILC3 NKp44$^-$ cells between HIV infected children and healthy controls. See Table S8 for full GSEA results; performed using the GO and KEGG terms available on MSigDB (v7.0).
