# Supplementary material for: Innate Lymphoid Cell Activation and Sustained Depletion in Blood and Tissue of Children Infected with HIV from Birth Despite Antiretroviral Therapy
Source: Cell Rep. 2020 Sep 15;32(11):108153. doi: 10.1016/j.celrep.2020.108153 (PMC7495043; doi:10.1016/j.celrep.2020.108153)
Supplement: Document S2. Article plus Supplemental Information [file mmc10.pdf]

Cell Reports

Article

Innate Lymphoid Cell Activation and Sustained Depletion in Blood and Tissue of Children Infected with HIV from Birth Despite Antiretroviral Therapy

Graphical Abstract

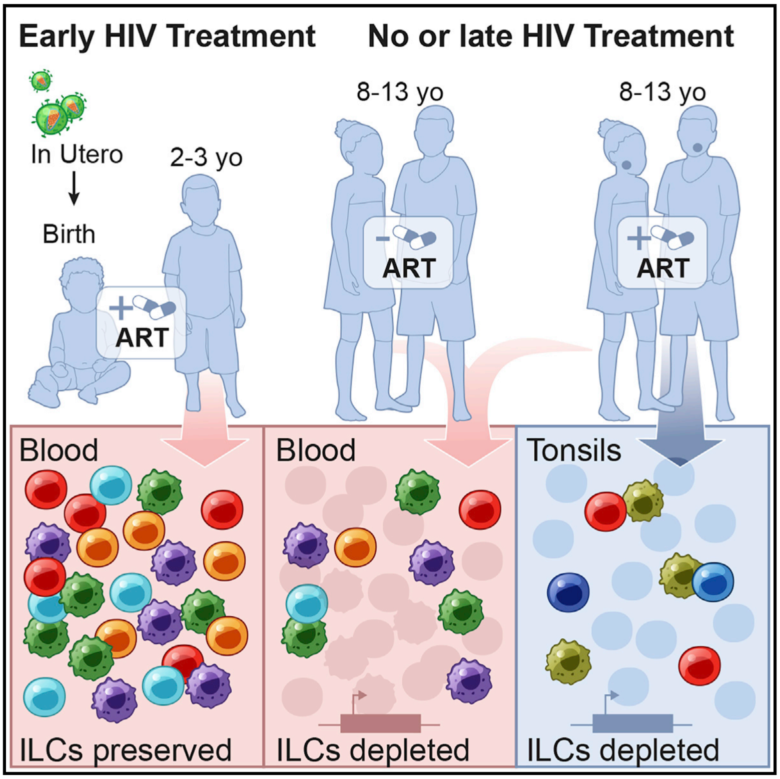

Highlights

- ILCs are depleted in children born with HIV-1 infection
- Only initiation of antiretroviral therapy at birth prevents ILC depletion
- Blood ILCs in HIV-1 infected children upregulate activation and cellular metabolism genes
- Tonsil-resident ILC3s and NK cells exhibit proliferation and innate immune signaling

Authors

Alveera Singh, Samuel W. Kazer, Julia Roider, ..., Philip Goulder, Alasdair Leslie, Henrik N. Kløverpris

Correspondence

henrik.kloverpris@ahri.org

In Brief

ILCs are dysregulated during HIV-1 infection in adults, but their fate in children is unknown. Singh et al. demonstrate that circulating and tonsil-resident ILCs are depleted in children infected with HIV-1 since birth. Transcriptionally, ILCs exhibit cell-type- and compartment-specific activity in HIV-1 infected children compared to healthy controls.

Singh et al., 2020, Cell Reports *32*, 108153
September 15, 2020 © 2020 The Author(s).
https://doi.org/10.1016/j.celrep.2020.108153

CellPress

Cell Reports

CellPress
OPEN ACCESS

Article

Innate Lymphoid Cell Activation and Sustained Depletion in Blood and Tissue of Children Infected with HIV from Birth Despite Antiretroviral Therapy

Alveera Singh,[1,15] Samuel W. Kazer,[2,3,4,15] Julia Roider,[1,5,7,8,15] Kami C. Krista,[2,3,4] Jane Millar,[5,7] Osaretin E. Asowata,[1] Abigail Ngoepe,[1] Duran Ramsuran,[1] Rabiah Fardoos,[1,9] Amanda Ardain,[1,14] Maximilian Muenchhoff,[5,11,12] Warren Kuhn,[6] Farina Karim,[1] Thumbi Ndung'u,[1,7,10,13] Alex K. Shalek,[2,3,4] Philip Goulder,[1,5,7] Alasdair Leslie,[1,10,14] and Henrik N. Kløverpris[1,9,10,14,16,*]

[1]Africa Health Research Institute (AHRI), Durban 4001, South Africa
[2]Institute for Medical Engineering and Science, Department of Chemistry, and Koch Institute for Integrative Cancer Research, Massachusetts Institute of Technology, Cambridge, MA 02139, USA
[3]Broad Institute of MIT and Harvard, Cambridge, MA 02139, USA
[4]Ragon Institute of MGH, Harvard, and MIT, Cambridge, MA 02139
[5]Department of Paediatrics, Peter Medawar Building for Pathogen Research, South Parks Rd, Oxford OX1 3SY, UK
[6]ENT Department General Justice Gizenga Mpanza Regional Hospital (Stanger Hospital), University of KwaZulu-Natal, Durban 4001, South Africa
[7]HIV Pathogenesis Programme, The Doris Duke Medical Research Institute, University of KwaZulu-Natal, Durban 4001, South Africa
[8]Medizinische Klinik IV, Department of Infectious Diseases, Ludwig-Maximilians-University, Munich 80802, Germany
[9]Department of Immunology and Microbiology, University of Copenhagen, Copenhagen 2200N, Denmark
[10]University College London, Division of Infection and Immunity, London WC1E 6AE, UK
[11]Max von Pettenkofer Institute, Virology, National Reference Center for Retroviruses, Faculty of Medicine, LMU München, Munich 81377, Germany
[12]German Center for Infection Research (DZIF), partner site Munich 80333, Germany
[13]Max Planck Institute for Infection Biology, Berlin 10117, Germany
[14]School of Laboratory Medicine and Medical Sciences, University of KwaZulu-Natal, Durban 4001, South Africa
[15]These authors contributed equally
[16]Lead Contact
*Correspondence: henrik.kloverpris@ahri.org
https://doi.org/10.1016/j.celrep.2020.108153

SUMMARY

Innate lymphoid cells (ILCs) are important for response to infection and for immune development in early life. HIV infection in adults depletes circulating ILCs, but the impact on children infected from birth remains unknown. We study vertically HIV-infected children from birth to adulthood and find severe and persistent depletion of all circulating ILCs that, unlike $CD4^+$ T cells, are not restored by long-term antiretroviral therapy unless initiated at birth. Remaining ILCs upregulate genes associated with cellular activation and metabolic perturbation. Unlike HIV-infected adults, ILCs are also profoundly depleted in tonsils of vertically infected children. Transcriptional profiling of remaining ILCs reveals ongoing cell-type-specific activity despite antiretroviral therapy. Collectively, these data suggest an important and ongoing role for ILCs in lymphoid tissue of HIV-infected children from birth, where persistent depletion and sustained transcriptional activity are likely to have long-term immune consequences that merit further investigation.

INTRODUCTION

In the absence of antiretroviral therapy (ART), HIV disease progression is typically more rapid in infected children compared to adults, with more than 50% mortality in HIV-infected children by 2 years of age (Goulder et al., 2016; Marston et al., 2011; Muenchhoff et al., 2014; Newell et al., 2004; Roider et al., 2016). In adult HIV infection, disease control is associated both with high $CD4^+$ T cell levels and low viral loads, as well as with strong HIV-specific adaptive immune responses (Goulder and Walker, 2012; Koup et al., 1994).

In pediatric HIV infection, however, only limited adaptive immunity operates to control viremia (E. Adland, 2014, IAS, conference; Adland et al., 2015; Muenchhoff et al., 2014). A subgroup of infected children (10%) maintain long-term HIV control that is associated with low immune activation of their T cells despite high viral loads (Muenchhoff et al., 2016; Roider et al., 2016). These individuals, termed ‘‘pediatric slow progressors’’ (PSPs), share several immunological features with the sooty mangabey natural hosts of simian immunodeficiency virus (SIV) infection, including modulation of type I interferon (IFN) pathways (Mandl et al., 2008; Meier et al., 2009) rather than strong HIV-specific

This is an open access article under the CC BY license (http://creativecommons.org/licenses/by/4.0/).

T cell responses linked to HIV control in adults (Chahroudi and Silvestri, 2016). In addition, non-pathogenic SIV of African green monkeys is associated with the migration of natural killer (NK) cells into lymphoid follicles through membrane-bound IL-15 on dendritic cells (Huot et al., 2017). This suggests innate lymphoid cell (ILC) subsets are involved in the prevention of disease in tissue, and these cells may play an important role in AIDS resistance in PSPs as well as in the natural hosts of SIV infection.

ILCs are a heterogenous group of lymphoid cells that do not express rearranged antigen receptors and therefore do not respond directly to antigen presentation (Vivier et al., 2018). They are grouped into helper and cytotoxic ILCs (NK cells) with functions and transcription factor expression overlapping that of $CD4^+$ T-helper cells and cytotoxic $CD8^+$ T cells, respectively. In the blood of HIV-infected children, NK cell expansion and activation occurs during the first year of life (Slyker et al., 2012) but declines with age irrespective of ART-mediated viral suppression (Azzoni et al., 2005); these NK cells appear to become dysfunctional (Ballan et al., 2007). Indeed, uninfected children of HIV-infected mothers display higher NK cell killing activities in the first 6 months of life compared to HIV-unexposed children (Smith et al., 2017). These studies suggest that NK cells in blood respond to HIV exposure and infection from birth. However, the impact of lifelong HIV infection on NK cells in lymphoid tissue, where they exert their effector function, remains unknown.

Helper ILCs are sentinels of infection at tissue sites where they are involved in maintaining homeostasis and repair after injury or infection (Shah et al., 2017). For example, we recently showed that ILCs are enriched in the human lung during active tuberculosis infection and are important for the early recruitment of macrophages (Ardain et al., 2019). Non-human primate studies of SIV infection show that ILCs display elevated apoptosis and cytotoxic phenotypes and are depleted in the gut (Klatt et al., 2012; Reeves et al., 2011), oral mucosae (Li and Reeves, 2013), and lymph nodes (Xu et al., 2015). In acute adult HIV infection, we observed irreversible ILC depletion from the blood unless treatment was initiated during early acute stages (Kløverpris et al., 2016). Subsequently, the loss of ILCs was shown to be directly linked to HIV-induced inflammatory cytokines (Wang et al., 2020). ILCs can respond to HIV infection at mucosal tissue sites (Fernandes et al., 2018; Kim et al., 2012) through type I IFN pathways (Wang et al., 2020) and FAS-FASL-induced apoptosis (Zhang et al., 2015) and are associated with markers of gut barrier breakdown and reduced IL-7 levels (Krämer et al., 2017), suggesting that ILCs are involved in tissue homeostasis during adult HIV infection (Shah et al., 2017). Interestingly, however, we found no depletion of ILCs from the tonsils or lungs of HIV-infected adults (Ardain et al., 2019; Kløverpris et al., 2016), suggesting that depletion of resident ILCs across tissues is not universal in adult HIV infection.

ILC composition is determined during early life in direct response to intestinal commensal microbial colonization (Gury-BenAri et al., 2016; Sonnenberg et al., 2012) and is regulated by the maternal microbiota (Gomez de Agüero et al., 2016) and maternal HIV exposure (Bender et al., 2016). Therefore, we hypothesized that lifelong HIV infection might have a different impact on circulating and tissue-resident ILCs than in adults. We studied a total of 229 newborns (NBs), children, and adults and mapped the circulating and tissue-resident ILC and NK cell response to HIV infection from birth; we observed striking ILC depletion in both the blood and tonsil tissue of infected children. Moreover, we found distinct cell-type-specific transcriptional changes in activation and metabolism, suggesting a potentially important role for innate lymphocytes in response to HIV infection in early life.

RESULTS

Helper ILCs in Blood Display a Distinct Transcriptional Profile Compared to NK Cells and Are Enriched in Children Compared to Adults

To evaluate the role of ILCs in pediatric HIV infection, we first established a gating strategy to analyze ILCs and NK cells in blood based on distinct phenotype expression (Lim et al., 2017; Spits et al., 2013). We excluded $lineage^+$ cells and used ILC- and NK-specific markers to simultaneously identify three different ILC (ILC1, ILC2, ILC pre-cursers [ILCPs]) (Lim et al., 2017) and two different NK cell populations (NK $CD56^{high}$ and NK $CD16^{high}$) (Figure 1A). We performed RNA sequencing (RNA-seq) of sorted ILC2, ILCP, NK $CD56^{high}$, and NK $CD16^{high}$ populations from 10 pediatric participants (median age 10.8 years, interquartile range [IQR] 6.4–11.9 years; Table S1). Principal component analysis (PCA) using 497 differentially expressed genes (DEGs) (false discovery rate [FDR]-corrected $q < 0.01$; see Method Details) demonstrated clean separation of these ILC subsets (Figure 1B; Table S2). Genes separating the subsets include granzyme B (*GZMB*), IFN-γ (*IFNG*), CD16 (*FCGR3A*), and *KIR2DL1* expressed in NK populations; and high levels of *KLRB1* (CD161), *KLRG1*, *CCR4*, *IL9R*, and *IL1RL1* (ST2), which binds IL-33 for activation in ILC2s (Figure 1C; Table S2). Thus, our flow cytometry panel successfully identifies the main ILC and NK cell subsets in pediatric blood, which also display the canonical gene signatures observed in adults.

Because the relative frequencies of many blood immune subsets change across the course of the normal lifespan (Prendergast et al., 2012; Shearer et al., 2003), we first studied ILC and NK levels in HIV-uninfected individuals spanning birth, childhood, and adulthood, in each case using samples from sub-Saharan African cohorts in Durban, South Africa (Table 1). Overall, among 138 HIV-uninfected individuals with an age range of 0–24 years, we found a strong reduction in the frequency of all ILC subsets with age (Figure 1D), whereas NK cell populations remained relatively stable (Figure 1E), consistent with a recent study (Vély et al., 2016). Together, these data define the circulating ILC populations present in children from sub-Saharan Africa and establish their normal frequencies in the absence of HIV infection.

Depletion of All Circulating ILC Subsets in Treatment-Naive HIV-Infected Children Irrespective of Disease Control

Mortality among vertically HIV-infected children exceeds 50% by the age of 2 years in the absence of ART (Marston et al., 2011; Newell et al., 2004). Here, we studied a group of 26 untreated vertically transmitted children that survived to the age of minimum 5 years in two different groups: (1) PSPs (median

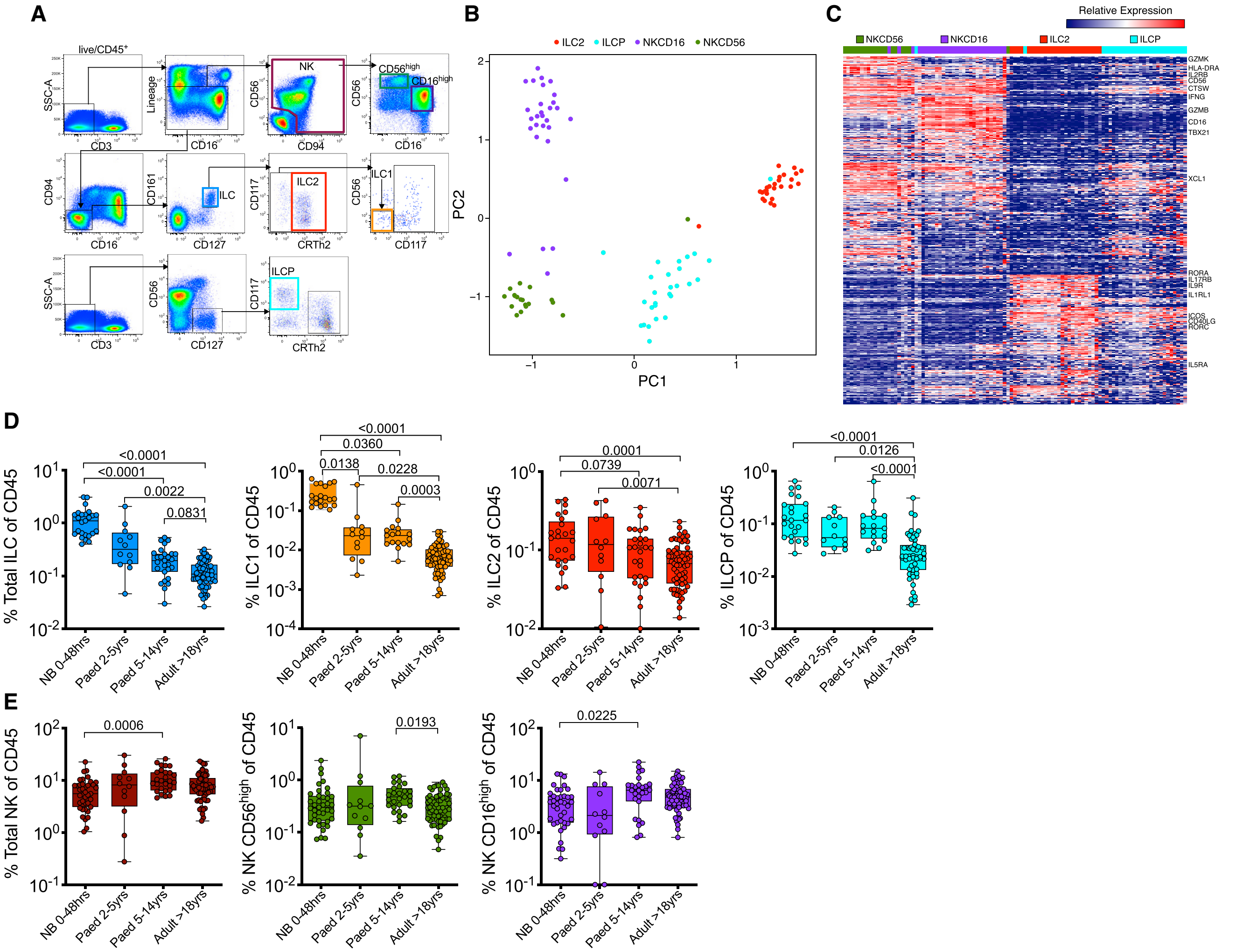

Figure 1. Circulating ILC Populations Decrease during the Course of Immune Maturation

(A) Gating strategy including lineage markers (CD3, CD4, CD11c, CD14, CD19, CD34, CD303, TCRγδ, TCRαβ) to identify two dominant NK populations defined by CD56^high^(green) and CD16^high^ (purple) and three ILC subsets: ILC1 (orange), ILC2 (red), and ILCP (light blue).

(B) Principal component analysis (PCA) and heatmap shown for each replicate for each participant (see Table S1).

(C) DEGs among ILC2, ILCP, CD56^{high} (NKCD56), and CD16^{high} (NKCD16) NK cell populations from four HIV-negative and six HIV-positive pediatric subjects.

(D) Frequencies of total helper ILC subsets as defined in (A), comparing HIV-negative newborn (NB) (n = 39), pediatric (2–5 years, n = 12), pediatric (>5 years, n = 25), and adult (n = 62) individuals expressed as percentage of total CD45^{+} lymphocytes.

(E) As in (C) but showing frequencies of total NK and subset-specific differences between pediatric and adult subjects.

p values by Dunn's multiple comparisons test.

CD4 = 37%, IQR 28%–44%, n = 15) and (2) pediatric progressors (PPs) (median CD4 = 15%, IQR 8%–28%, n = 11) who met World Health Organization (WHO) criteria prevailing at the time of the study to initiate antiretroviral treatment (Table 1; Figure 2A). Total ILC frequencies and all individual helper ILC subsets (ILC1, ILC2, and ILCPs) were significantly decreased compared to uninfected controls. However, there were no significant differences between PSPs and PPs (Figure 2B). Overall, the NK populations showed a similar pattern of depletion, though mostly found within the CD56^{high} NK subset ($p < 0.0002$) (Figure 2C). Thus, all helper ILCs and NK cells are severely depleted in children infected with HIV at birth, even among the rare group of PSPs, who maintain normal-for-age CD4 levels (Figure 2A) despite being ART naive.

ILC Depletion in the Blood Is Sustained Despite Viral Suppression by ART but Can be Prevented by Immediate ART Treatment Initiation at Birth

We have previously reported that long-term ART in chronic adult infection is unable to restore circulating ILC levels (Kløverpris et al., 2016). However, HIV-infected children appear to possess a superior ability to restore adaptive immune function compared to adults (Lewis et al., 2012; Picat et al., 2013). Therefore, we next investigated the impact of long-term ART in pediatric HIV

Table 1. Clinical Characteristics of 229 Newborn, Pediatric, and Adult Subjects

Cohort	HIV Transmission	n	Age[a]	ART	Weeks on ART	CD4%	Viral Load (VL)[b]
Newborns HIV^{-}	none	39	23 (20–30) (h)	NA	NA	51 (43–54)	NA
Infants HIV^{-}	none	12	48 (36–57) (min)	NA	NA	32 (7–43)	NA
Infants HIV^{+}	*in utero*	27	21 (12–24) (min)	yes	79 (51–103)	30 (26–35)	<20
Pediatric HIV^{-}	none	25	8.8 (7.0–12.0) (years)	NA	NA	37 (28–44)	NA
Pediatric HIV^{+} PSP	vertical	15	12.6 (11.0–14.1) (years)	no	NA	30 (22–37)	9,500 (1,450–53,000)
Pediatric HIV^{+} PP	vertical	11	7.6 (6.8–12.7) (years)	no	NA	15 (8–28)	110,000 (21,000–510,000)
Pediatric HIV^{+} PART	vertical	38	11.9 (8.6–15.2) (years)	yes	88 (40–218)	30 (19–36	<20
Adult HIV uninfected	none	62	21 (20–22) (years)	NA	NA	41 (37–45)	NA

HIV^{-}, HIV uninfected; HIV^{+}, HIV infected; ART, antiretroviral therapy; NA, not applicable
[a]Median
[b]HIV RNA copies/ml plasma

infection in a cohort of children (n = 38) treated for a median of 88 weeks (IQR 40–218) (Table 1). As in adult HIV infection, ART failed to restore total ILCs, ILC1, ILC2, ILCPs, and NK cells (Figure 3A). Longitudinal sampling of the PP cohort (n = 9) over three time points from a median of 12 weeks before treatment and again at 42 and 84 weeks after treatment initiation showed the same trend. Although a modest increase in both NK CD56^{high} cells and ILCPs was observed, neither of which reached levels of uninfected controls (Figure 3B). This lack of ILC or NK cell reconstitution was confirmed in additional longitudinal cohorts undergoing viral suppression by ART or maintaining high CD4 levels in the absence of treatment over 84 weeks (Figure S1).

Our previous work demonstrated that early treatment of HIV-infected adults, prior to peak viremia, prevents significant loss of circulating ILCs (Kløverpris et al., 2016). To test this, we next examined the effect of early treatment initiation on circulating ILC subsets in a cohort of 27 *in-utero*-infected newborns (NBs), initiated on ART within minutes after birth (Adland et al., 2020). ART was initially prophylactic (nevirapine and zidovudine) and then increased to triple therapy treatment from a median of 7 days post-partum (range 0–18 days), with all individuals remaining undetectable for plasma HIV RNA at 21 months of age (Table 1). Strikingly, in these individuals, we found no difference in CD4^{+} T cell, ILC, or NK cell levels compared to age-matched HIV-uninfected children (Figure 3C). Thus, immediate ART initiation of *in-utero*-infected NBs preserves ILC and NK cell levels, which is consistent with that seen in adult horizontal HIV infection (Kløverpris et al., 2016).

Peripheral ILCs and NK Cells Are Transcriptionally Activated in the Blood of HIV-Infected Children

To investigate whether blood ILCs in children are modulated by HIV infection, we measured surface *ex vivo* activation marker expression CD69 and FAS (CD95) and cytokine production (IL-2, IL-4, IL-5, IL-13) following mitogen stimulation on ILC2, ILCP, NK CD56^{high}, and NK CD16^{high} subsets. HIV had no impact on expression of CD69 and Fas on ILC2s and ILCPs, while NK CD56^{high} and NK CD16^{high} subsets displayed increased expression of Fas and CD69 (Munneke et al., 2014) (Figure S2A), indicating that they are stimulated by HIV infection. In the mitogen stimulation assay of ILC2s, we found no impact of HIV infection on cytokine production (Figure S2B).

To further explore the impact of HIV on ILCs in circulation in infected children, we performed RNA-seq on ILC2, ILCP, NK CD56^{high}, and NK CD16^{high} subsets and CD4^{+} T cells from three ART-naive viremic PSPs, three ART-treated virally suppressed children, and four age-matched HIV-uninfected children (see Table S1 for replicate numbers and Method Details). Differential gene expression analyses on each subset between HIV-infected children (PSP^{+} ART$^{+/-}$) and HIV-uninfected controls demonstrated >300 DEGs (FDR-corrected q < 0.1) in each of the CD4^{+} T cells, ILC2s, ILCPs, and NK CD16^{high} cells and 61 DEGs in NK CD56^{high} cells (Figure 4A; Table S3). Comparisons between both virally suppressed and viremic HIV-infected children and between virally suppressed HIV-infected children and HIV-uninfected controls revealed few transcriptional changes in all cell subsets (<57 and <49, respectively), suggesting that persistent viremia drives transcriptional changes in circulating ILCs (Figure 4B; Table S3).

Analysis of the DEGs observed in all subsets is consistent with the activation and loss of regulatory function of ILC subsets in chronically HIV-infected children. We note the following DEGs in each subset comparing HIV-infected children to healthy controls (Figure 4C; Table S3): (1) ILC2s: downregulation of inflammatory markers *PTGS2*, *NFKBID*, and *TNF* and upregulation of anti-apoptosis marker *BIRC3*, H4 clustered histone 3 (*HIST1H4C*), and *SUB1,* a transcription factor that mediates RNA polymerase binding; (2) ILCPs: downregulation of ILC3 lineage transcription factor *RORC* (Serafini et al., 2015) and upregulation of pro-survival markers (*AXL*, *MET*) and cytokine-induced transcription factor *STAT4*; (3) NK CD16^{high}: downregulation of the mTORC-activating factor *KLHL22* (Chen et al., 2018) and upregulation of *TNFRSF9* (CD137), an activation marker of NK cells (Baessler et al., 2010) known to promote T cell expansion (Wilcox et al., 2002), and *GK* encoding glycerol kinase, an essential enzyme for glycerol conversion; and (4) NK CD56^{high}: downregulation of canonical transcription factors involved in negative regulation of cellular proliferation and differentiation (*DUSP6, FOS*) and upregulation of *MORC3*, which recruits p53 and other transcription machinery (Sloan et al., 2016; Takahashi et al., 2007), and *IFI30*, an IFN-stimulated response gene. Moreover, genes involved in active metabolism like COX7A2, NDUFB6 (ILC2), NDUFA12 (ILCP), ACSL1 (NK CD16^{high}), and DNAJA4 (NK CD56^{high}) were upregulated in HIV-infected children,

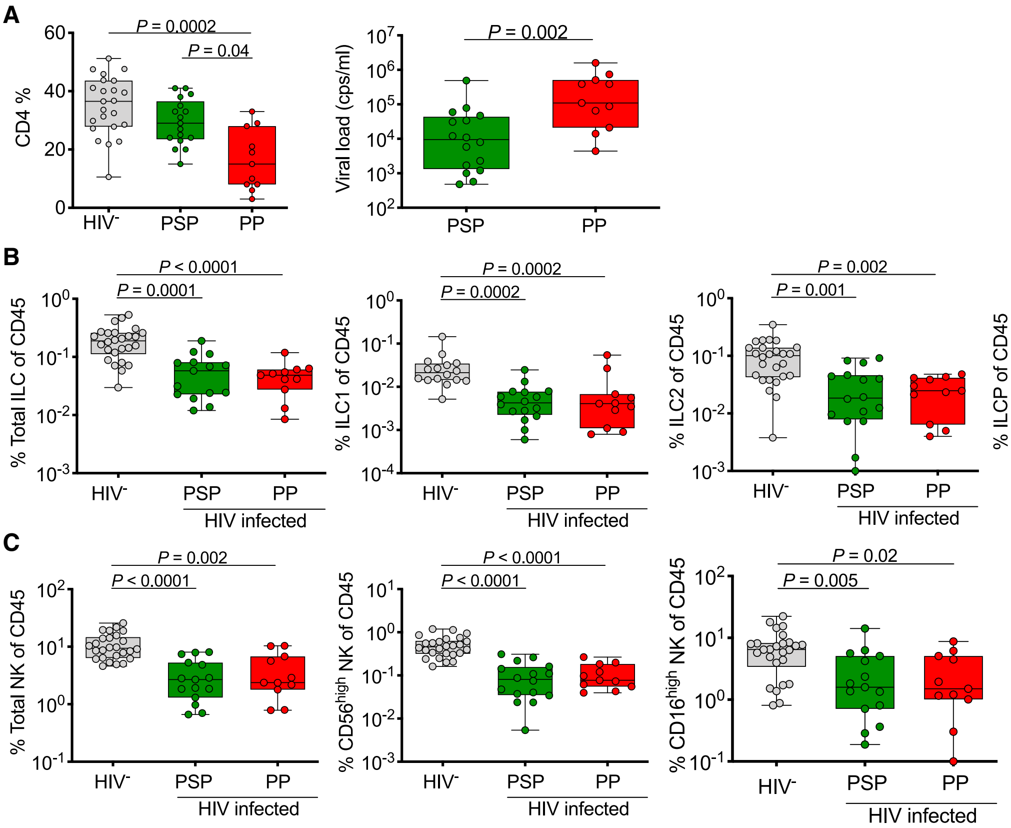

Figure 2. Depletion of Peripheral Helper ILC and Cytotoxic NK Cell Subsets in Treatment-Naive HIV-Infected Pediatric Subjects

(A) CD4 percentage of $CD45^+$ lymphocytes (left) and plasma viral load (right) of pediatric HIV-uninfected (HIV^-) subjects (n = 25), pediatric slow progressors (PSPs) (n = 15), and pediatric progressors (PPs) (n = 11) (see Table 1).

(B) As in (A) but showing total ILC (left), ILC1 (center left), ILC2 (center right), and ILCP (right) subset levels as percentage of $CD45^+$ lymphocytes. Gating as in Figure 1A.

(C) As in (B) but showing total NK cells (left) and NK subsets ($CD56^{high}$, middle; $CD16^{high}$, right) as percentage of $CD45^+$ lymphocytes.

p values by Dunn's multiple comparisons test.

suggesting active metabolism in these subsets in response to infection.

Gene set analysis using ingenuity pathway analysis (IPA) highlighted oncogenes ESR1, RET, and MYC as significantly enriched and upregulated upstream drivers potentially inducing the transcriptional changes in all ILC subsets (except for ESR1 in ILC2s and MYC in NK $CD16^{high}$ cells) between HIV-infected children and healthy controls (Figure 4D; Table S4). Consistent with a role for IFN in chronic HIV infection (Roff et al., 2014), IFN-γ is also significantly enriched, though lacking in ILC2s. These upstream drivers are corroborated by enrichment for genes associated with protein ubiquitination in all ILC subsets in downstream pathway analysis (Figure 4E; Table S4). Surprisingly, both ILC2s and ILCPs exhibit significant enrichment for pathways annotated for defects in cellular metabolism—mitochondrial dysfunction and unfolded protein response, respectively. To confirm these metabolic gene signatures, we performed gene set enrichment analysis (GSEA) on the DEGs from each ILC subset using the Gene Ontology (GO) and KEGG databases (see Method Details). In both ILC2s and ILCPs, several GO and KEGG terms encompassing cellular activation and metabolism were positively enriched (Figure 2C; Table S4). GSEA on NK $CD16^{high}$ DEGs implicated pathogen recognition receptor signaling and response to stress, while no terms were significant for enrichment from NK $CD56^{high}$ DEGs (Method Details). Together, these data demonstrate that peripheral ILC2s, ILCPs, NK $CD16^{high}$, and NK $CD56^{high}$ subsets all express activation gene programming in HIV-infected children compared to HIV-uninfected controls. Moreover, enrichment results suggest differences in cellular metabolism in ILC2s and ILCPs.

Reduced ILC and NK Levels in Tonsils from HIV-Infected Children

While the role and function of helper ILCs in the blood are unknown, ILCs play a key role in human lymphoid tissue development (Koues et al., 2016; Vély et al., 2016) and in response to inflammation (Bernink et al., 2013). Using tonsils from children undergoing tonsillectomy as a source of secondary lymphoid tissue (Table S5) (Roider et al., 2019), we identified six different innate lymphocyte populations from lineage-negative cells (Figure S3A), which were dominated by NK cells

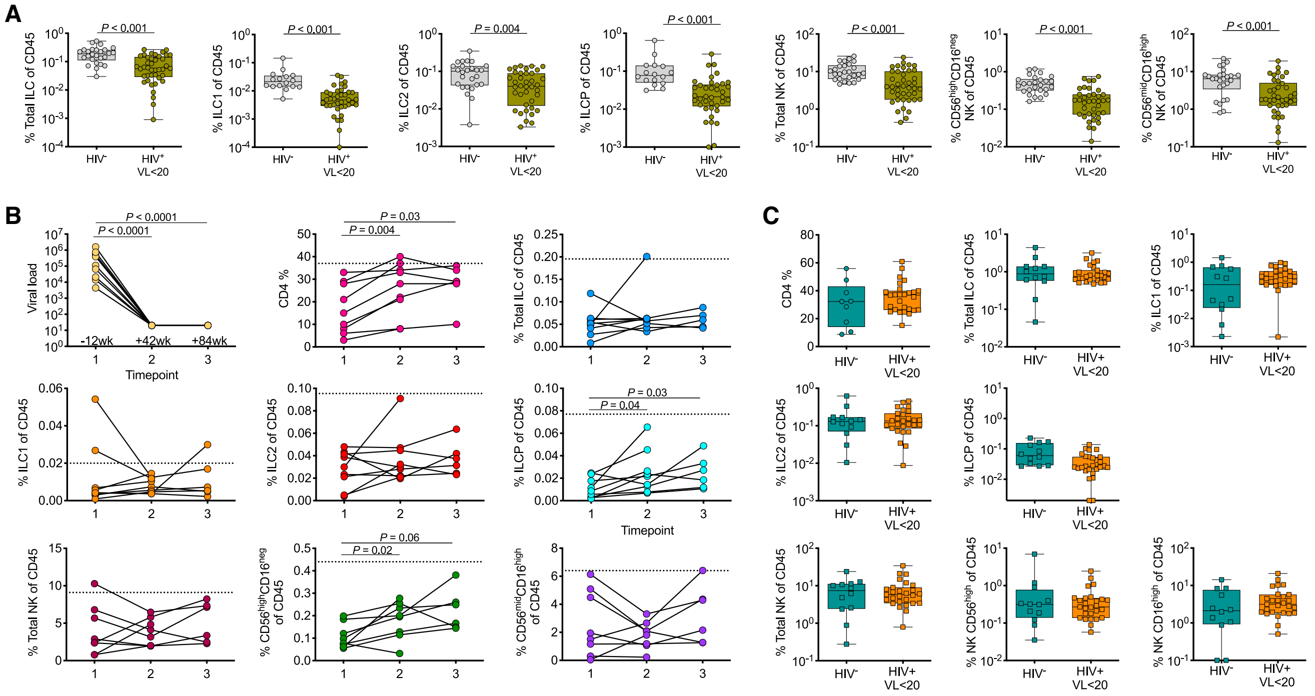

Figure 3. Sustained Depletion of All Peripheral ILC and NK Subsets in Virally Suppressed Children in the Absence of Treatment Initiation at Birth

(A) Cross-sectional comparisons of all blood helper ILC and NK subsets in pediatric HIV-uninfected and virally suppressed (VL <20 HIV RNA copies/ml plasma) pediatric subjects treated for a median of 88 weeks (IQR 40–218) with median CD4% of 37 (IQR 26–35) and 30 (IQR 19–36), respectively.

(B) Longitudinal sampling of pediatric subjects (n = 9) before treatment initiation and at two time points after treatment intervention: time point 1 = 12 weeks before starting ART, time point 2 = 42 weeks after treatment, and time point 3 = 84 weeks after treatment when patients have fully suppressed plasma viral loads and reconstituted CD4 percentages (see also Figure S1). The dotted lines represent normal levels of HIV-negative pediatric subjects.

(C) Blood CD4, helper ILC, and NK subset percentages in HIV-uninfected infants aged 2–5 years (infant HIV$^-$; n = 12; petrol) and HIV$^+$ and viral-suppressed (VL <20 HIV RNA copies/ml plasma) infants aged 0.2–3 years (infant HIV$^+$; n = 27; orange).

p values by Dunn's multiple comparisons test.

and ILC3s (Figures S3B and S3C). We found differential expression of CD103 and CD69, described as surrogate markers for tissue residency (Masopust and Soerens, 2019; Skon et al., 2013) (Figure 5A). Overall, CD127$^-$ NK cells and NKp44$^+$ ILC3s expressed higher levels of these markers of tissue residence compared to ILC1, ILC2, and CD127$^+$ NK cells, consistent with mouse model experiments (Gasteiger et al., 2015) (Figure 5B). To test if tonsil-resident ILCs also were reduced by HIV infection, we compared the relative frequency of each of the tonsil NK and ILC subsets from 12 HIV-negative children to that of 4 ART-treated HIV-positive children in whom the ART initiation was unknown, of which 3 were virally suppressed (viral load <20 copies/ml plasma) with detectable antiretroviral drugs in plasma (Table S5). We found highly significant depletion for each ILC and NK subset (Figure 5C) but, surprisingly, no significant depletion of bulk CD4$^+$ T cells or PD-1+$^+$CD69$^+$ T-follicular helper-like cells in the same subjects (Figure S3D), in contrast to our previous work in adult tonsils where we found no HIV-associated ILC depletion (Kløverpris et al., 2016). Although the number of HIV-infected children studied here is small, these data suggest that HIV infection from birth has a more severe impact on tissue-resident ILCs than does infection in later life.

Transcriptional Profiling of NK and ILC3 Cells in Tonsils from HIV-Infected Children Reveals Subset-Specific Activation in Response to Infection

Next, to study the ILC and NK cell responses to HIV infection in pediatric tissue, we purified the dominant ILC (ILC3 NKp44$^-$, ILC3 NKp44$^+$) and NK cell subsets (NK CD127$^-$) from pediatric tonsils, while insufficient cell numbers were available from ILC1 and ILC2 subsets (see Figure S3A), and performed transcriptional profiling directly *ex vivo* to characterize both the transcriptional differences between the subsets and their responses to HIV infection (Figure 6). In HIV-uninfected controls, a limited number of genes separated the two ILC3 subsets defined by NKp44 surface expression (238 DEGs, FDR-corrected q < 0.1) compared to >1,000 genes for both ILC3 subsets compared to NK CD127$^-$ cells (Figure 6A; Table S6). In the top 20 regulated genes between ILC3 subsets were *NCR2*, which encodes NKp44, and *S1PR1* (CD69), which is associated with tissue residency (Skon et al., 2013); both are consistent with protein surface expression on ILC3 subsets (see Figure 5 and Figure S3A). Comparison of the NK CD127$^-$ and ILC3 subsets showed strong differential expression of canonical genes known to be upregulated in ILC3s (*ICAM1*, *ICOS*, *IL1R1*, *RORC*, *IL17RE*, *KIT*, *ICOS*, *CD83*) whereas NK CD127$^-$ cells expressed genes

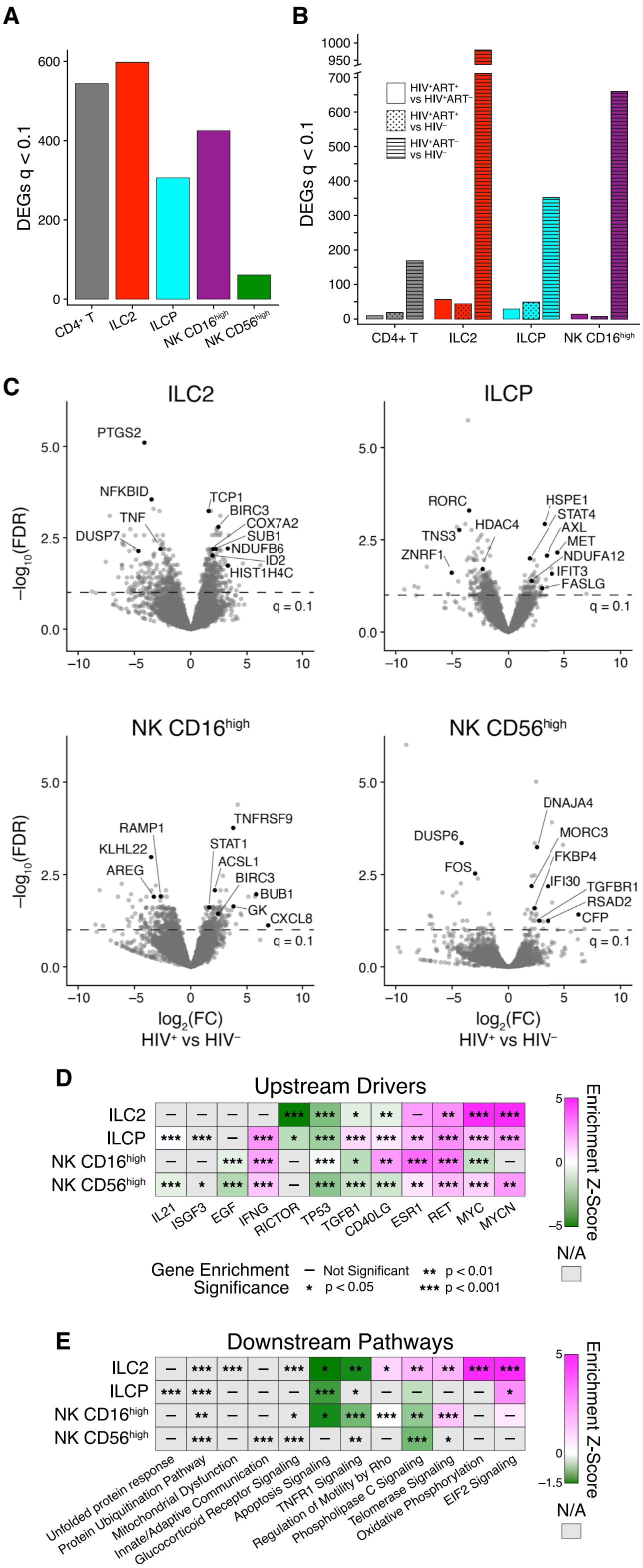

Figure 4. Blood ILC Subsets Are Transcriptionally Activated during Chronic Pediatric HIV

(A and B) Number of DEGs in whole transcriptomes of CD4+ T cells, ILC2s, ILCPs, CD56high, and CD16high NK cells between HIV-infected and HIV-uninfected pediatric subjects (A) and among HIV-uninfected, HIV-infected treated and HIV-infected untreated pediatric subjects (B) (see Table S1 for subject numbers). DEGs were called using DESeq2 with a significance cut-off of FDR < 0.1.

(C) Volcano plots of the DEGs between HIV-infected (positive) and HIV-uninfected (negative) pediatric subjects in (A). Genes of interest are annotated with black dots; see Table S3 for all DEGs. Dotted line annotates the significance cut-off of FDR *q* < 0.1.

(D and E) Select upstream drivers (D) and canonical pathways (E) significant in ingenuity pathway analysis (IPA) of DEGs from each ILC subset. For directionally annotated pathways, a *Z*-score is calculated to represent up- or downregulation of the driver or pathway. If a driver or pathway is not directionally annotated in IPA, or there are not enough genes in the list to calculate a *Z*-score, N/A is reported. See Table S4 for the full IPA results. See also Figure S2.

involved in cytotoxicity (*GZMA*, *GZMB*, *CCL5*, *GNLY*, *PRF1*, *IFNG*) together with expression of canonical NK cell surface molecules (*FCGR3A*, *KLRF1*, *KLRG1*). In addition, we found ILC3-specific upregulation of genes associated with homing to lymphoid follicular zones (*CXCR5*, *CXCR4*, *CCR6*) and regulation of adaptive immunity (*ICOS*, *CD40L*, *IFNGR2*). These data are consistent with previous analysis in human tonsils (Björklund et al., 2016; Cella et al., 2019; Koues et al., 2016) and suggest that ILC3 and NK subsets have distinct functions in human lymphoid tissue as regulators of tissue homeostasis and killing potential, respectively.

To determine the response of each of these subsets to HIV infection in children, we compared the transcriptional response in ILC3 NKp44$^-$, ILC3 NKp44$^+$, and NK CD127$^-$ cells in tonsils from two virally suppressed ART-treated HIV-infected children with five age- and gender- (female) matched HIV-uninfected children (see Table S6). We found 195, 286, and 75 DEGs (FDR-corrected q < 0.1) for ILC3 NKp44$^-$, ILC3 NKp44$^+$, and NK CD127$^-$ cells, respectively (Figure 6B; Table S7). We note the following DEGs in each subset comparing HIV-infected children to healthy controls (Figure 6C; Table S7): (1) ILC3 NKp44$^-$: upregulation of NF-κB co-activator *PRMT6* (Di Lorenzo et al., 2014), cell-cycle antigen *PCNA*, and *CD38*, which is associated with activation in T cells, but previously undescribed on ILCs; (2) ILC3 NKp44$^+$: downregulation of NF-κB inhibitor *MAST2* (Xiong et al., 2004) and upregulation of cell-cycle-associated *TUBB2A*, anti-inflammatory *ANXA1* (Arcone et al., 1993), and lineage commitment protein *ID1*, whose family member ID2 is known to regulate ILC differentiation in tissue (Zhong and Zhu, 2017); and (3) NK CD127$^-$: upregulation of secreted pattern recognition receptor lectin *CLEC18A* shown to be associated with hepatitis C infection and Notch signaling pathway genes *JAG2* and *NOD2*. Notch signaling has been shown to upregulate killer immunoglobulin-like receptor (KIR) expression and drive maturation in NK cells (Felices et al., 2014), suggesting that this NK CD127$^-$ subset may play an extended effector role in the tonsil in HIV infection.

To examine the CD4+ T cell response to HIV infection in pediatric tonsils, we sorted four different CD4+ T cell populations based on CD103/PD-1 expression (Figure S4A) accounting for

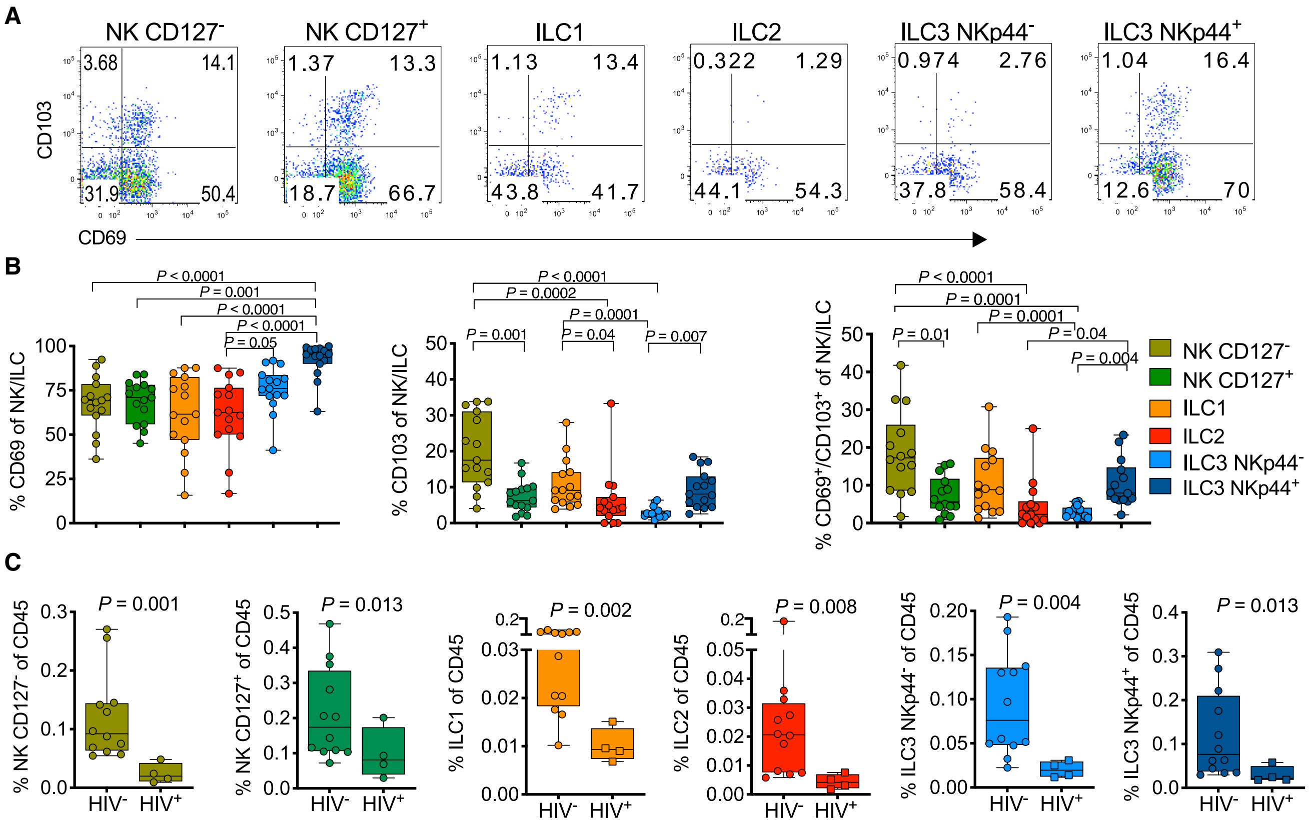

Figure 5. Lymphoid Tissue-Resident ILCs Are Reduced in the Tonsils of HIV-Infected Children

(A) Gating of CD69 and CD103 co-expression from each of the six innate subsets as indicated above flow plots (see Figure S3).

(B) Frequencies of CD69 (left), CD103 (center), and combined CD69/CD103 (right) expression on six innate tonsil NK/ILC cell populations as defined in (A) in 15 pediatric subjects. p values by Dunn's multiple comparisons test.

(C) Frequencies of each of the six innate lymphocyte population in tonsils, comparing pediatric HIV-uninfected (HIV$^-$; n = 12) and HIV-infected (HIV$^+$; n = 4) subjects. (See also Figure S3). P values by Mann-Whitney U test.

the majority of CD4$^+$ T cells with distinct gene expression to that of innate lymphocytes (Table S7). The PD-1^{++}CD103$^-$ subset expressed high levels of the canonical T follicular helper (Tfh) cell genes *CXCR5* and *PDCD1* and low levels of *ITGAE* (CD103), reflecting the sorted phenotypes by flow (Figure S4B). Using samples collected from a separate set of children given limited sample availability (see Table S5), we compared gene expression in each CD4$^+$ T cell subset between HIV-infected (n = 3) and HIV-uninfected (n = 5) children (see Method Details for design). We found limited numbers of DEGs in all four CD4 T cell subsets (Figure S4C; Table S7), suggesting limited response from CD4$^+$ T cells compared to that seen for ILCs and NK cells.

Gene set analysis distinguished the responses to HIV infection by subset, with unique potential upstream drivers and pathways significantly enriched in each (Figures 6D and 6E; Table S7). While the ILC3 NKp44$^-$ subset expressed DEGs associated with innate and cytokine-mediated immune signaling potentially induced by broad transcriptional activator SP1 and epigenetic regulator HDAC3, the enrichment on the DEGs in the ILC3 NKp44$^+$ subset reflected general cellular activation and proliferation with potential activation by G-coupled protein receptors and nuclear transcription factor HFN4A. Enrichment on the NK CD127$^-$ subset demonstrated a consistent role for NK cells in the tonsil as innate immune sentinels during HIV infection, with putative upstream driver PTPRM, known to regulate cellular adhesion. GSEA using the KEGG and GO gene sets found significant enrichment only in the ILC3 NKp44$^-$ subset, supporting broad cellular activation and proliferation with significant GO terms encompassing protein complex assembly, signal transduction by p53, and biogenesis (Figure S4D; Table S8).

In summary, the ILC and NK cells responses to pediatric HIV infection were cell-subset specific with distinct activation and proliferation programming. CD4$^+$ T cells in the tonsil, on the other hand, did not demonstrate strong transcriptional differences between HIV-infected and HIV-uninfected children. While the ILC3 NKp44$^+$ subset did not exhibit immune activation by flow cytometry, both the ILC3 NKp44$^-$ and NK CD127$^-$ subsets upregulated machinery associated with immune response and innate immune signaling. Together with the profound depletion of ILCs and NK cells observed in vertically infected children, these data suggest that HIV infection from birth has a persistent impact on innate lymphocytes within secondary lymphoid organs that may have important consequences for their downstream immune health.

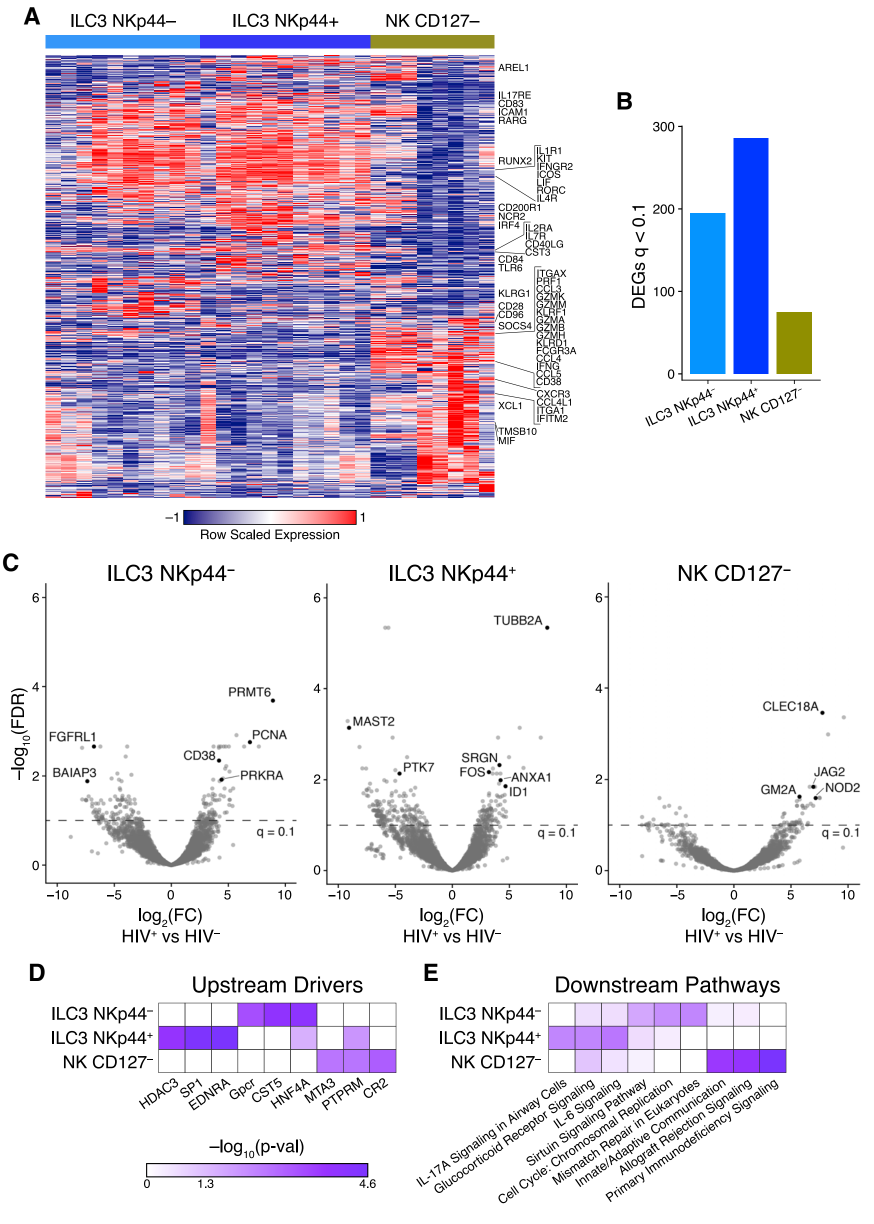

Figure 6. Coordinated Transcriptional ILC3 and NK Cell Response in the Pediatric HIV-Infected Tonsil

(A) Heatmap showing DEGs among ILC3 NKp44⁻, ILC3 NKp44⁺, and NK CD127⁻ subsets from four HIV-negative pediatric tonsils performed in duplicate or triplicates with canonical genes annotated.

(B) Number of DEGs in whole transcriptomes of tonsils of ILC3 NKp44⁻, ILC3 NKp44⁺, and NK CD127⁻ cells among viral-suppressed (<20 HIV RNA copies/ml plasma), HIV-infected (HIV⁺; n = 3), and gender- and age-matched HIV-uninfected pediatric subjects (HIV⁻; n = 4).

(C) Volcano plot showing significance plotted against $\log_2$ fold change for DEGs in the ILC3 NKp44⁻, ILC3 NKp44⁺, and NK CD127⁻ populations with genes upregulated in HIV-infected tonsils shown as positive $\log_2$ fold change (right) and genes downregulated by HIV infection shown as negative $\log_2$ fold change (left). Genes of interest are annotated with black points and with dotted line annotating the significance cut-off of FDR q < 0.1.

(D and E) Upstream molecules predicted to be involved in initiating pathways shown in (E) determined by ingenuity pathway analysis of DEGs from the each of the three innate tonsil lymphocyte subsets. See also Figure S4.

DISCUSSION

The past decade has established ILCs as key players in orchestrating tissue homeostasis and repair (Vivier et al., 2018). While HIV infection is known to cause irreversible changes in some human tissues (Deeks et al., 2013), the impact on ILC number and function remains incompletely understood, especially following vertical HIV infection in blood and tissue during early life. We hypothesized that innate lymphocyte responses may be particularly important in pediatric HIV infection while the adaptive immune response is still developing.

Here, we studied 229 individuals spanning the time from birth to adulthood and show that circulating ILCs are dramatically reduced in vertically HIV-infected individuals and are not restored by successful long-term ART unless it is initiated at birth. This is similar to our finding in adult HIV infection, in which only ART started in Fiebig stages I–VI was able to preserve circulating ILC levels (Kløverpris et al., 2016). However, unlike adult infection, in which tissue-resident ILCs with tonsils were preserved, vertical HIV infection also caused severe depletion of all ILC subsets within these secondary lymphoid organs. Remaining tissue-resident ILCs displayed diverse responses to HIV infection that involves proliferation, activation, and potential differentiation, consistent with immune signatures observed in lymph nodes from adult non-human primates and human subjects (Mudd et al., 2018; Wang et al., 2020). These data showing persistent transcriptional activity suggest an ongoing functional role for lymph node ILCs in pediatric HIV infection. The severe depletion of these cells, therefore, seems likely to have long consequences to the lymph node function and immune health.

Using a gating strategy previously shown to identify circulating ILCs in adults (Kløverpris et al., 2016), we identify all known ILC subsets within our pediatric subsets and confirmed four of these subsets at the transcriptional level. Interestingly, we find that helper ILC levels, but not cytotoxic NK levels, are highly elevated in early life and decrease toward adolescence. This is consistent with recent data from cord blood and peripheral blood of pediatrics in Caucasian populations (Vély et al., 2016) and suggests

that helper ILCs may play a more dominant role in the immune response during early life while the adaptive immune response is maturing.

The cohorts spanning early life to adolescence show in each case that HIV infection reduces or depletes circulating helper ILCs and CD56^{high} NK cells in the absence of early ART intervention. In our treatment-naive cohorts, we stratified for disease control by comparing PSPs (Muenchhoff et al., 2016) and PPs but found no overall differences in ILC subsets between these otherwise clinically distinct groups. PSPs are characterized by normal-to-age CD4 levels and very low immune activation despite high viremia, in contrast to progressing children (Muenchhoff et al., 2016). This does not agree with the findings of Mudd et al. (2018), who found a direct correlation between ILCs and CD4 levels in individuals. Importantly, however, that association was observed in HIV-infected adults and in individuals with non-HIV-associated reduced CD4 counts. This study shows that ILC depletion in the context of vertical HIV infection, however, is clearly not directly related to CD4 counts. Indeed, our findings may question the mechanistic relationship between CD4 count and circulating ILCs, as it is clearly not a dependent relationship. Moreover, virally suppressed pediatric individuals followed longitudinally showed no reconstitution of helper ILCs or CD56^{high} NK cells despite normalization of CD4 levels. Consistent with this, ILC levels in adolescents (14–18 years) remained low despite >4 years of treatment. Alternative mechanisms suggest HIV induced cytokines to drive ILC depletion (Wang et al., 2020), although this was not investigated here.

While the importance of ILCs in tissue is now well established (Vivier et al., 2018), ILCs' function in blood remains unknown. Our transcriptional profiling of blood ILCs showed robust transcriptional responses for helper ILCs and NK cell subsets consisting of DEGs associated with broad cellular activation. The fact that IFN-γ was predicted as an important upstream driver of this response (except in ILC2s) is consistent with a pervasive role for this cytokine in orchestrating peripheral immune responses during chronic HIV infection (Roff et al., 2014). In adult HIV infection, apoptotic signatures were detected in acute HIV infection and associated with ILC depletion (Kløverpris et al., 2016), consistent with recent work in showing HIV-induced cytokines can deplete ILC homeostasis in adult HIV infection (Wang et al., 2020). However, no such apoptotic signature was detected in the HIV-infected pediatric subjects studied here, who were not in the acute phase. Indeed, upregulation of the antiapoptotic factor *BIRC3* in ILC2s and *AXL* and *MET* in ILCPs was observed. We also observed significant upregulation of genes enriched for metabolic pathways in ILC2s and ILCPs. Recent work in healthy tissue has demonstrated that ILCs play an important role in regulating dietary and tissue metabolism, and changes in the cellular metabolism of ILCs can affect the immunoregulatory effects of these cells (O'Sullivan and Sun, 2017; Wilhelm et al., 2017). Thus, although this study lacks mechanistic detail, this gene modulation is consistent with published data and may suggest a role for ILCs in the immunometabolic effects of HIV infection. In the remaining ILCs sequenced from HIV-infected tonsils, we did indeed detect an upregulation of genes involved in metabolism. However, we do observe a clear signature of immune-related gene networks, including signaling and tissue repair. Surprisingly, we observed little impact from HIV infection on CD4^{+} T cell subsets, including Tfh cells, in pediatric tonsils. Direct matched comparison of CD4^{+} T cell subsets between blood and tonsils is needed to confirm this difference in transcriptional response by compartment. Although further work is needed, these data imply an ongoing role of ILCs within secondary lymphoid organs of HIV-infected children. Whether these are protective or detrimental to lymph node function in these individuals remains to be seen.

In conclusion, we demonstrate the impact of lifelong HIV infection on ILCs in both blood and lymphoid tissue. We used well-defined cohorts differentiated by relative natural disease control and time to treatment initiation at birth and early childhood. Despite their functional overlap to helper CD4^{+} T cells, it is clear that ILCs in blood respond differently in both frequencies and function to HIV infection and to antiretroviral HIV treatment. Moreover, ILC responses at tissue effector sites point toward a role for these cells as important regulators of tissue homeostasis in chronic treated HIV infection. Properly functioning lymph nodes are crucial for the generation of optimal immune responses, and it is known that even treated HIV-infected children have impaired immune responses to both vaccination (Cagigi et al., 2012) and natural infection (Muenchhoff et al., 2019). ILCs are required for the formation of secondary lymphoid organs during development (van de Pavert et al., 2014) and their proper functioning (Bar-Ephraïm and Mebius, 2016). Thus, the depletion of ILCs observed in children with lifelong HIV infection may contribute to suboptimal immunity in these individuals. Crucially, the consequences of HIV infection from birth in later life remain unknown. Understanding the impact of HIV-induced depletion of ILCs in lymph nodes may lead to interventions that improve immune function in this vulnerable and important population.

STAR★METHODS

Detailed methods are provided in the online version of this paper and include the following:

- KEY RESOURCES TABLE
- RESOURCE AVAILABILITY
  - Lead contact
  - Materials availability
  - Data and code availability
- EXPERIMENTAL MODEL AND SUBJECT DETAILS
- METHOD DETAILS
  - Cell isolation from human blood and tonsil
  - Flow cytometry analysis and cell sorting
  - RNA isolation, library construction, sequencing, and alignment
  - RNA-Seq Differential Expression Analysis
  - Gene Set Analysis
- QUANTIFICATION AND STATISTICAL ANALYSIS

SUPPLEMENTAL INFORMATION

Supplemental Information can be found online at https://doi.org/10.1016/j.celrep.2020.108153.

ACKNOWLEDGMENTS

We wish to thank all the mother-child pairs, children, adolescents, and adults participating in this study. We also want to thank all the staff at the Human Pathogenesis Programme (HPP) and at the Africa Health Research Institute (AHRI), and associated medical and hospital clinical staff at Ithembalabantu clinic, Edendale, Mahatma Gandhi Memorial, and Stanger and Queen Nandi Memorial Hospitals in KZN. H.N.K. was supported by The Wellcome Trust (202485/Z/16/Z) and the Maersk Foundation for Medical Improvements. A.L. is supported by The Wellcome Trust (210662/Z/18/Z). A.K.S. was supported, in part, by the Searle Scholars Program, the Beckman Young Investigator Program, the National Institutes of Health (NIH) (5U24AI118672, 2R01HL095791, 2U19AI089992, 1R01HL134539,1R01AI138546), a Sloan Fellowship in Chemistry, and the Bill and Melinda Gates Foundation. S.W.K. was supported by the Hugh Hampton Young Memorial Fellowship. P.G. was supported by the NIH (R01 AI133673) and the Wellcome Trust (WT104748MA). This work was supported through the Sub-Saharan African Network for TB/HIV Research Excellence (SANTHE), a Developing Excellence in Leadership, Training and Science (DELTAS) Africa Initiative (DEL-15-006). The DELTAS Africa Initiative is an independent funding scheme of the African Academy of Sciences (AAS)'s Alliance for Accelerating Excellence in Science in Africa (AESA) and is supported by the New Partnership for Africa's Development Planning and Coordinating Agency (NEPAD Agency) with funding from The Wellcome Trust (107752/Z/15/Z) and the UK Government. The views expressed in this publication are those of the author(s) and not necessarily those of AAS, NEPAD Agency, the Wellcome Trust, or the UK government. H.N.K. and A.S. were supported by the Sub-Saharan African Network for TB/HIV Research Excellence (SANTHE).

AUTHOR CONTRIBUTIONS

A.S. and J.R. performed experiments. S.W.K. and K.K. performed transcriptional analysis. J.R., J.M., and M.M. contributed human samples. O.E.A., A.N., D.R., R.F., and A.A. contributed to experimental work. W.K. contributed surgical human tissues samples. F.K. and T.N. contributed sample collections. A.K.S. supervised data analysis. P.G. and A.L. provided clinical samples. S.W.K., A.L., and H.N.K. prepared the manuscript. H.N.K. conceptualized. S.W.K., A.K.S., P.G., A.L., and H.N.K. provided intellectual input.

DECLARATION OF INTERESTS

The authors declare no competing interests.

Received: August 5, 2019
Revised: March 14, 2020
Accepted: August 25, 2020
Published: September 15, 2020

REFERENCES

Adland, E., Paioni, P., Thobakgale, C., Laker, L., Mori, L., Muenchhoff, M., Csala, A., Clapson, M., Flynn, J., Novelli, V., et al. (2015). Discordant Impact of HLA on Viral Replicative Capacity and Disease Progression in Pediatric and Adult HIV Infection. PLoS Pathog. *11*, e1004954.

Adland, E., Millar, J., Bengu, N., Muenchhoff, M., Fillis, R., Sprenger, K., Ntlantsana, V., Roider, J., Vieira, V., Govender, K., et al. (2020). Sex-specific innate immune selection of HIV-1 in utero is associated with increased female susceptibility to infection. Nat. Commun. *11*, 1767.

Arcone, R., Arpaia, G., Ruoppolo, M., Malorni, A., Pucci, P., Marino, G., Ialenti, A., Di Rosa, M., and Ciliberto, G. (1993). Structural characterization of a biologically active human lipocortin 1 expressed in Escherichia coli. Eur. J. Biochem. *211*, 347–355.

Ardain, A., Domingo-Gonzalez, R., Das, S., Kazer, S.W., Howard, N.C., Singh, A., Ahmed, M., Nhamoyebonde, S., Rangel-Moreno, J., Ogongo, P., et al. (2019). Group 3 innate lymphoid cells mediate early protective immunity against tuberculosis. Nature *570*, 528–532.

Azzoni, L., Rutstein, R.M., Chehimi, J., Farabaugh, M.A., Nowmos, A., and Montaner, L.J. (2005). Dendritic and natural killer cell subsets associated with stable or declining CD4+ cell counts in treated HIV-1-infected children. J. Infect. Dis. *191*, 1451–1459.

Baessler, T., Charton, J.E., Schmiedel, B.J., Grünebach, F., Krusch, M., Wacker, A., Rammensee, H.G., and Salih, H.R. (2010). CD137 ligand mediates opposite effects in human and mouse NK cells and impairs NK-cell reactivity against human acute myeloid leukemia cells. Blood *115*, 3058–3069.

Ballan, W.M., Vu, B.A., Long, B.R., Loo, C.P., Michaëlsson, J., Barbour, J.D., Lanier, L.L., Wiznia, A.A., Abadi, J., Fennelly, G.J., et al. (2007). Natural killer cells in perinatally HIV-1-infected children exhibit less degranulation compared to HIV-1-exposed uninfected children and their expression of KIR2DL3, NKG2C, and NKp46 correlates with disease severity. J. Immunol. *179*, 3362–3370.

Bar-Ephraïm, Y.E., and Mebius, R.E. (2016). Innate lymphoid cells in secondary lymphoid organs. Immunol. Rev. *271*, 185–199.

Bender, J.M., Li, F., Martelly, S., Byrt, E., Rouzier, V., Leo, M., Tobin, N., Pannaraj, P.S., Adisetiyo, H., Rollie, A., et al. (2016). Maternal HIV infection influences the microbiome of HIV-uninfected infants. Sci. Transl. Med. *8*, 349ra100.

Bernink, J.H., Peters, C.P., Munneke, M., te Velde, A.A., Meijer, S.L., Weijer, K., Hreggvidsdottir, H.S., Heinsbroek, S.E., Legrand, N., Buskens, C.J., et al. (2013). Human type 1 innate lymphoid cells accumulate in inflamed mucosal tissues. Nat. Immunol. *14*, 221–229.

Björklund, A.K., Forkel, M., Picelli, S., Konya, V., Theorell, J., Friberg, D., Sandberg, R., and Mjösberg, J. (2016). The heterogeneity of human CD127(+) innate lymphoid cells revealed by single-cell RNA sequencing. Nat. Immunol. *17*, 451–460.

Cagigi, A., Cotugno, N., Giaquinto, C., Nicolosi, L., Bernardi, S., Rossi, P., Douagi, I., and Palma, P. (2012). Immune reconstitution and vaccination outcome in HIV-1 infected children: present knowledge and future directions. Hum. Vaccin. Immunother. *8*, 1784–1794.

Cella, M., Gamini, R., Secca, C., Collins, P.L., Zhao, S., Peng, V., Robinette, M.L., Schettini, J., Zaitsev, K., Gordon, W., et al. (2019). Subsets of ILC3-ILC1-like cells generate a diversity spectrum of innate lymphoid cells in human mucosal tissues. Nat. Immunol. *20*, 980–991.

Chahroudi, A., and Silvestri, G. (2016). What pediatric nonprogressors and natural SIV hosts teach us about HIV. Sci. Transl. Med. *8*, 358fs16.

Chen, J., Ou, Y., Yang, Y., Li, W., Xu, Y., Xie, Y., and Liu, Y. (2018). KLHL22 activates amino-acid-dependent mTORC1 signalling to promote tumorigenesis and ageing. Nature *557*, 585–589.

Deeks, S.G., Tracy, R., and Douek, D.C. (2013). Systemic effects of inflammation on health during chronic HIV infection. Immunity *39*, 633–645.

Di Lorenzo, A., Yang, Y., Macaluso, M., and Bedford, M.T. (2014). A gain-of-function mouse model identifies PRMT6 as a NF-κB coactivator. Nucleic Acids Res. *42*, 8297–8309.

Felices, M., Ankarlo, D.E., Lenvik, T.R., Nelson, H.H., Blazar, B.R., Verneris, M.R., and Miller, J.S. (2014). Notch signaling at later stages of NK cell development enhances KIR expression and functional maturation. J. Immunol. *193*, 3344–3354.

Fernandes, S.M., Pires, A.R., Matoso, P., Ferreira, C., Nunes-Cabaço, H., Correia, L., Valadas, E., Poças, J., Pacheco, P., Veiga-Fernandes, H., et al. (2018). HIV-2 infection is associated with preserved GALT homeostasis and epithelial integrity despite ongoing mucosal viral replication. Mucosal Immunol. *11*, 236–248.

Gasteiger, G., Fan, X., Dikiy, S., Lee, S.Y., and Rudensky, A.Y. (2015). Tissue residency of innate lymphoid cells in lymphoid and nonlymphoid organs. Science *350*, 981–985.

Gomez de Agüero, M., Ganal-Vonarburg, S.C., Fuhrer, T., Rupp, S., Uchimura, Y., Li, H., Steinert, A., Heikenwalder, M., Hapfelmeier, S., Sauer, U., et al. (2016). The maternal microbiota drives early postnatal innate immune development. Science *351*, 1296–1302.

Goulder, P.J., and Walker, B.D. (2012). HIV and HLA class I: an evolving relationship. Immunity *37*, 426–440.

Goulder, P.J., Lewin, S.R., and Leitman, E.M. (2016). Paediatric HIV infection: the potential for cure. Nat. Rev. Immunol. *16*, 259–271.

Gury-BenAri, M., Thaiss, C.A., Serafini, N., Winter, D.R., Giladi, A., Lara-Astiaso, D., Levy, M., Salame, T.M., Weiner, A., David, E., et al. (2016). The Spectrum and Regulatory Landscape of Intestinal Innate Lymphoid Cells Are Shaped by the Microbiome. Cell *166*, 1231–1246.e1213.

Huot, N., Jacquelin, B., Garcia-Tellez, T., Rascle, P., Ploquin, M.J., Madec, Y., Reeves, R.K., Derreudre-Bosquet, N., and Müller-Trutwin, M. (2017). Natural killer cells migrate into and control simian immunodeficiency virus replication in lymph node follicles in African green monkeys. Nat. Med. *23*, 1277–1286.

Kim, C.J., Nazli, A., Rojas, O.L., Chege, D., Alidina, Z., Huibner, S., Mujib, S., Benko, E., Kovacs, C., Shin, L.Y., et al. (2012). A role for mucosal IL-22 production and Th22 cells in HIV-associated mucosal immunopathogenesis. Mucosal Immunol. *5*, 670–680.

Klatt, N.R., Estes, J.D., Sun, X., Ortiz, A.M., Barber, J.S., Harris, L.D., Cervasi, B., Yokomizo, L.K., Pan, L., Vinton, C.L., et al. (2012). Loss of mucosal CD103+ DCs and IL-17+ and IL-22+ lymphocytes is associated with mucosal damage in SIV infection. Mucosal Immunol. *5*, 646–657.

Kløverpris, H.N., Kazer, S.W., Mjösberg, J., Mabuka, J.M., Wellmann, A., Ndhlovu, Z., Yadon, M.C., Nhamoyebonde, S., Muenchhoff, M., Simoni, Y., et al. (2016). Innate Lymphoid Cells Are Depleted Irreversibly during Acute HIV-1 Infection in the Absence of Viral Suppression. Immunity *44*, 391–405.

Koues, O.I., Collins, P.L., Cella, M., Robinette, M.L., Porter, S.I., Pyfrom, S.C., Payton, J.E., Colonna, M., and Oltz, E.M. (2016). Distinct Gene Regulatory Pathways for Human Innate versus Adaptive Lymphoid Cells. Cell *165*, 1134–1146.

Koup, R.A., Safrit, J.T., Cao, Y., Andrews, C.A., McLeod, G., Borkowsky, W., Farthing, C., and Ho, D.D. (1994). Temporal association of cellular immune responses with the initial control of viremia in primary human immunodeficiency virus type 1 syndrome. J. Virol. *68*, 4650–4655.

Krämer, B., Goeser, F., Lutz, P., Glässner, A., Boesecke, C., Schwarze-Zander, C., Kaczmarek, D., Nischalke, H.D., Branchi, V., Manekeller, S., et al. (2017). Compartment-specific distribution of human intestinal innate lymphoid cells is altered in HIV patients under effective therapy. PLoS Pathog. *13*, e1006373.

Lewis, J., Walker, A.S., Castro, H., De Rossi, A., Gibb, D.M., Giaquinto, C., Klein, N., and Callard, R. (2012). Age and CD4 count at initiation of antiretroviral therapy in HIV-infected children: effects on long-term T-cell reconstitution. J. Infect. Dis. *205*, 548–556.

Li, B., and Dewey, C.N. (2011). RSEM: accurate transcript quantification from RNA-Seq data with or without a reference genome. BMC Bioinformatics *12*, 323.

Li, H., and Reeves, R.K. (2013). Functional perturbation of classical natural killer and innate lymphoid cells in the oral mucosa during SIV infection. Front. Immunol. *3*, 417.

Lim, A.I., Li, Y., Lopez-Lastra, S., Stadhouders, R., Paul, F., Casrouge, A., Serafini, N., Puel, A., Bustamante, J., Surace, L., et al. (2017). Systemic Human ILC Precursors Provide a Substrate for Tissue ILC Differentiation. Cell *168*, 1086–1100.e1010.

Love, M.I., Huber, W., and Anders, S. (2014). Moderated estimation of fold change and dispersion for RNA-seq data with DESeq2. Genome Biology *15*. https://doi.org/10.1186/s13059-014-0550-8.

Mandl, J.N., Barry, A.P., Vanderford, T.H., Kozyr, N., Chavan, R., Klucking, S., Barrat, F.J., Coffman, R.L., Staprans, S.I., and Feinberg, M.B. (2008). Divergent TLR7 and TLR9 signaling and type I interferon production distinguish pathogenic and nonpathogenic AIDS virus infections. Nat. Med. *14*, 1077–1087.

Marston, M., Becquet, R., Zaba, B., Moulton, L.H., Gray, G., Coovadia, H., Essex, M., Ekouevi, D.K., Jackson, D., Coutsoudis, A., et al. (2011). Net survival of perinatally and postnatally HIV-infected children: a pooled analysis of individual data from sub-Saharan Africa. Int. J. Epidemiol. *40*, 385–396.

Masopust, D., and Soerens, A.G. (2019). Tissue-Resident T Cells and Other Resident Leukocytes. Annu. Rev. Immunol. *37*, 521–546.

Meier, A., Chang, J.J., Chan, E.S., Pollard, R.B., Sidhu, H.K., Kulkarni, S., Wen, T.F., Lindsay, R.J., Orellana, L., Mildvan, D., et al. (2009). Sex differences in the Toll-like receptor-mediated response of plasmacytoid dendritic cells to HIV-1. Nat. Med. *15*, 955–959.

Mudd, J.C., Busman-Sahay, K., DiNapoli, S.R., Lai, S., Sheik, V., Lisco, A., Deleage, C., Richardson, B., Palesch, D.J., Paiardini, M., et al. (2018). Hallmarks of primate lentiviral immunodeficiency infection recapitulate loss of innate lymphoid cells. Nat. Commun. *9*, 3967.

Muenchhoff, M., Prendergast, A.J., and Goulder, P.J. (2014). Immunity to HIV in Early Life. Front. Immunol. *5*, 391.

Muenchhoff, M., Adland, E., Karimanzira, O., Crowther, C., Pace, M., Csala, A., Leitman, E., Moonsamy, A., McGregor, C., Hurst, J., et al. (2016). Nonprogressing HIV-infected children share fundamental immunological features of nonpathogenic SIV infection. Sci. Transl. Med. *8*, 358ra125.

Muenchhoff, M., Adland, E., Roider, J., Kløverpris, H., Leslie, A., Boehm, S., Keppler, O.T., Ndung'u, T., and Goulder, P.J.R. (2019). Differential Pathogen-Specific Immune Reconstitution in Antiretroviral Therapy-Treated Human Immunodeficiency Virus-Infected Children. J. Infect. Dis. *219*, 1407–1417.

Munneke, J.M., Björklund, A.T., Mjösberg, J.M., Garming-Legert, K., Bernink, J.H., Blom, B., Huisman, C., van Oers, M.H., Spits, H., Malmberg, K.J., and Hazenberg, M.D. (2014). Activated innate lymphoid cells are associated with a reduced susceptibility to graft-versus-host disease. Blood *124*, 812–821.

Newell, M.L., Coovadia, H., Cortina-Borja, M., Rollins, N., Gaillard, P., and Dabis, F.; Ghent International AIDS Society (IAS) Working Group on HIV Infection in Women and Children (2004). Mortality of infected and uninfected infants born to HIV-infected mothers in Africa: a pooled analysis. Lancet *364*, 1236–1243.

O'Sullivan, T.E., and Sun, J.C. (2017). Innate Lymphoid Cell Immunometabolism. J. Mol. Biol. *429*, 3577–3586.

Picat, M.Q., Lewis, J., Musiime, V., Prendergast, A., Nathoo, K., Kekitiinwa, A., Nahirya Ntege, P., Gibb, D.M., Thiebaut, R., Walker, A.S., et al.; ARROW Trial Team (2013). Predicting patterns of long-term CD4 reconstitution in HIV-infected children starting antiretroviral therapy in sub-Saharan Africa: a cohort-based modelling study. PLoS Med. *10*, e1001542.

Prendergast, A.J., Klenerman, P., and Goulder, P.J. (2012). The impact of differential antiviral immunity in children and adults. Nat. Rev. Immunol. *12*, 636–648.

Reeves, R.K., Rajakumar, P.A., Evans, T.I., Connole, M., Gillis, J., Wong, F.E., Kuzmichev, Y.V., Carville, A., and Johnson, R.P. (2011). Gut inflammation and indoleamine deoxygenase inhibit IL-17 production and promote cytotoxic potential in NKp44+ mucosal NK cells during SIV infection. Blood *118*, 3321–3330.

Roff, S.R., Noon-Song, E.N., and Yamamoto, J.K. (2014). The Significance of Interferon-γ in HIV-1 Pathogenesis, Therapy, and Prophylaxis. Front. Immunol. *4*, 498.

Roider, J.M., Muenchhoff, M., and Goulder, P.J. (2016). Immune activation and paediatric HIV-1 disease outcome. Curr. Opin. HIV AIDS *11*, 146–155.

Roider, J., Porterfield, J.Z., Ogongo, P., Muenchhoff, M., Adland, E., Groll, A., Morris, L., Moore, P.L., Ndung'u, T., Kløverpris, H., et al. (2019). Plasma IL-5 but Not CXCL13 Correlates With Neutralization Breadth in HIV-Infected Children. Front. Immunol. *10*, 1497.

Serafini, N., Vosshenrich, C.A., and Di Santo, J.P. (2015). Transcriptional regulation of innate lymphoid cell fate. Nat. Rev. Immunol. *15*, 415–428.

Shah, S.V., Manickam, C., Ram, D.R., and Reeves, R.K. (2017). Innate Lymphoid Cells in HIV/SIV Infections. Front. Immunol. *8*, 1818.

Shearer, W.T., Rosenblatt, H.M., Gelman, R.S., Oyomopito, R., Plaeger, S., Stiehm, E.R., Wara, D.W., Douglas, S.D., Luzuriaga, K., McFarland, E.J., et al.; Pediatric AIDS Clinical Trials Group (2003). Lymphocyte subsets in healthy children from birth through 18 years of age: the Pediatric AIDS Clinical Trials Group P1009 study. J. Allergy Clin. Immunol. *112*, 973–980.

CellPress
OPEN ACCESS

Skon, C.N., Lee, J.Y., Anderson, K.G., Masopust, D., Hogquist, K.A., and Jameson, S.C. (2013). Transcriptional downregulation of S1pr1 is required for the establishment of resident memory CD8+ T cells. Nat. Immunol. *14*, 1285–1293.

Sloan, E., Orr, A., and Everett, R.D. (2016). MORC3, a Component of PML Nuclear Bodies, Has a Role in Restricting Herpes Simplex Virus 1 and Human Cytomegalovirus. J. Virol. *90*, 8621–8633.

Slyker, J.A., Lohman-Payne, B., John-Stewart, G.C., Dong, T., Mbori-Ngacha, D., Tapia, K., Atzberger, A., Taylor, S., Rowland-Jones, S.L., and Blish, C.A. (2012). The impact of HIV-1 infection and exposure on natural killer (NK) cell phenotype in Kenyan infants during the first year of life. Front. Immunol. *3*, 399.

Smith, C., Jalbert, E., de Almeida, V., Canniff, J., Lenz, L.L., Mussi-Pinhata, M.M., Cohen, R.A., Yu, Q., Amaral, F.R., Pinto, J., et al. (2017). Altered Natural Killer Cell Function in HIV-Exposed Uninfected Infants. Front. Immunol. *8*, 470.

Sonnenberg, G.F., Monticelli, L.A., Alenghat, T., Fung, T.C., Hutnick, N.A., Kunisawa, J., Shibata, N., Grunberg, S., Sinha, R., Zahm, A.M., et al. (2012). Innate lymphoid cells promote anatomical containment of lymphoid-resident commensal bacteria. Science *336*, 1321–1325.

Spits, H., Artis, D., Colonna, M., Diefenbach, A., Di Santo, J.P., Eberl, G., Koyasu, S., Locksley, R.M., McKenzie, A.N., Mebius, R.E., et al. (2013). Innate lymphoid cells–a proposal for uniform nomenclature. Nat. Rev. Immunol. *13*, 145–149.

Subramanian, A., Tamayo, P., Mootha, V.K., Mukherjee, S., Ebert, B.L., Gillette, M.A., Paulovich, A., Pomeroy, S.L., Golub, T.R., Lander, E.S., and Mesirov, J.P. (2005). Gene set enrichment analysis: a knowledge-based approach for interpreting genome-wide expression profiles. Proc. Natl. Acad. Sci. USA *102*, 15545–15550.

Takahashi, K., Yoshida, N., Murakami, N., Kawata, K., Ishizaki, H., Tanaka-Okamoto, M., Miyoshi, J., Zinn, A.R., Shime, H., and Inoue, N. (2007). Dynamic regulation of p53 subnuclear localization and senescence by MORC3. Mol. Biol. Cell *18*, 1701–1709.

van de Pavert, S.A., Ferreira, M., Domingues, R.G., Ribeiro, H., Molenaar, R., Moreira-Santos, L., Almeida, F.F., Ibiza, S., Barbosa, I., Goverse, G., et al. (2014). Maternal retinoids control type 3 innate lymphoid cells and set the offspring immunity. Nature *508*, 123–127.

Vély, F., Barlogis, V., Vallentin, B., Neven, B., Piperoglou, C., Ebbo, M., Perchet, T., Petit, M., Yessaad, N., Touzot, F., et al. (2016). Evidence of innate lymphoid cell redundancy in humans. Nat. Immunol. *17*, 1291–1299.

Vivier, E., Artis, D., Colonna, M., Diefenbach, A., Di Santo, J.P., Eberl, G., Koyasu, S., Locksley, R.M., McKenzie, A.N.J., Mebius, R.E., et al. (2018). Innate Lymphoid Cells: 10 Years On. Cell *174*, 1054–1066.

Wang, Y., Lifshitz, L., Gellatly, K., Vinton, C.L., Busman-Sahay, K., McCauley, S., Vangala, P., Kim, K., Derr, A., Jaiswal, S., et al. (2020). HIV-1-induced cytokines deplete homeostatic innate lymphoid cells and expand TCF7-dependent memory NK cells. Nat. Immunol. *21*, 274–286.

Wilcox, R.A., Tamada, K., Strome, S.E., and Chen, L. (2002). Signaling through NK cell-associated CD137 promotes both helper function for CD8+ cytolytic T cells and responsiveness to IL-2 but not cytolytic activity. J. Immunol. *169*, 4230–4236.

Wilhelm, C., Kharabi Masouleh, S., and Kazakov, A. (2017). Metabolic Regulation of Innate Lymphoid Cell-Mediated Tissue Protection-Linking the Nutritional State to Barrier Immunity. Front. Immunol. *8*, 1742.

Xiong, H., Li, H., Chen, Y., Zhao, J., and Unkeless, J.C. (2004). Interaction of TRAF6 with MAST205 regulates NF-kappaB activation and MAST205 stability. J. Biol. Chem. *279*, 43675–43683.

Xu, H., Wang, X., Lackner, A.A., and Veazey, R.S. (2015). Type 3 innate lymphoid cell depletion is mediated by TLRs in lymphoid tissues of simian immunodeficiency virus-infected macaques. FASEB J. *29*, 5072–5080.

Zhang, Z., Cheng, L., Zhao, J., Li, G., Zhang, L., Chen, W., Nie, W., Reszka-Blanco, N.J., Wang, F.S., and Su, L. (2015). Plasmacytoid dendritic cells promote HIV-1-induced group 3 innate lymphoid cell depletion. J. Clin. Invest. *125*, 3692–3703.

Zhong, C., and Zhu, J. (2017). Transcriptional regulators dictate innate lymphoid cell fates. Protein Cell *8*, 242–254.

STAR★METHODS

KEY RESOURCES TABLE

REAGENT or RESOURCE	SOURCE	IDENTIFIER
Antibodies		
Anti-CD11c AF488 (Lineage)	BioLegend	301618; RRID: AB_439791
Anti-CD14 FITC (Lineage)	BD Bioscience	555397; RRID: AB_395798
Anti-CD19 FITC (Lineage)	BD Bioscience	560994; RRID: AB_10563406
Anti-CD3 AF488 (Lineage)	BioLegend	300320; RRID: AB_493691
Anti-CD4 FITC (Lineage)	BioLegend	317420; RRID: AB_571939
Anti-TCRgd AF488 (Lineage)	BioLegend	331208; RRID: AB_1575108
Anti-TCRab AF488 (Lineage)	BioLegend	306712; RRID: AB_528967
Anti-CD34 FITC (Lineage)	BioLegend	343604; RRID: AB_1732005
Anti-CD303 FITC (Lineage)	BioLegend	354208; RRID: AB_2561364
Anti-CD19 FITC (Lineage)	BD Bioscience	560994; RRID: AB_10563406
Anti-CD94 PerCp.Cy5.5	BD Bioscience	562361; RRID: AB_11152081
Anti-CD117 BV421	BioLegend	313216; RRID: AB_11148721
Anti-CD117 BV650	BioLegend	313221; RRID: AB_2562714
Anti-CD161 BV605	BioLegend	339915; RRID: AB_11142679
Anti-CD16 BV650	BioLegend	302042; RRID: AB_2563801
Anti-CD56 BV711	BioLegend	318336; RRID: AB_2562417
Anti-CD3 BV785	BioLegend	317330; RRID: AB_2563507
Anti-CD294 AF647	BD Bioscience	558042; RRID: AB_2112699
Anti-CD38 AF700	BioLegend	303516; RRID: AB_2072782
Anti-CD95 PE-CF594	BioLegend	305634; RRID: AB_2564221
Anti-CD127 Pe-Cy7	Beckman Coulter	A14934; RRID: AB_2534372
Anti-CD4 BUV496	BD Bioscience	564651; RRID: AB_2744422
Anti-PD-1 BV421	BD Bioscience	562516; RRID: AB_11153482
Anti-CD103 BV605	BioLegend	350218; RRID: AB_2564283
Anti-CD69 BV785	BioLegend	310932; RRID: AB_2563696
Anti-CD3 PE-CF594	BD Bioscience	562280; RRID: AB_11153674
Anti-CD366 (NKp44) PE-Cy5	Beckman Coulter	A66903; RRID: AB_2857937
Anti-CD8 BUV396	BD Bioscience	563795; RRID: AB_2722501
Anti-CD19 BUV496	BD Bioscience	564655; RRID: AB_2744311
Live/Dead Fixable Near-IR Dead Cell Stain Kit, 633nm	Invitrogen	L10119
Anti-IL-2 BV650	BD Biosciencecces	563467; RRID: AB_2738224
Anti-IL-4 FITC	BioLegend	500807; RRID: AB_315126
Anti-IL-5 APC	BioLegend	504305; RRID: AB_315329
Anti-IL-13 BV421	BioLegend	561158; RRID: AB_10561838
Anti-TNFa AFlour700	BD Bioscience	557996; RRID: AB_396978
Anti-IFNg PE-Cy7	BD Bioscience	557643; RRID: AB_396760
Anti-CD294 (CTRH2) PE-CF594	BD Bioscience	563501; RRID: AB_2738244
Anti-CD127 PE-Cy5	Beckman Coulter	A64617; RRID: AB_2833010
Biological Samples		
Human peripheral blood mononuclear cells (PBMCs)	Human	Cohorts
Human tonsil mononuclear cells (TMCs)	Human	Cohorts
(See Table 1)		

(*Continued on next page*)

Continued

REAGENT or RESOURCE	SOURCE	IDENTIFIER
Chemicals, Peptides, and Recombinant Proteins		
Maxima H-RT and Buffer	ThermoFisher Scientific	EP0751
dNTPs	New England Biolabs	N0447L
SUPERase*In RNase inhibitor	ThermoFisher Scientific	AM2696
Betaine solution, 5M, PCR Reagent	Millipore Sigma	B0300-5VL
KAPA 2x HiFi HotStart PCR mix	Kapa Biosystems	KK2602
RNAClean XP	Beckman Coulter	A63987
AMPure XP	Beckman Coulter	A63881
Nextera XT Kit	Illumina, Inc	FC-131-1096
Oligonucleotides		
SMART Oligo dT	IDT	/5Biosg/AAGCAGTGGTATCAACGCAGAGTAC$(T)_{30}$VN
Template-Switching Oligo	IDT	AAGCAGTGGTATCAACGCAGAGTACATrGrGrG
SMART PCR Primer	IDT	AAGCAGTGGTATCAACGCAGAGT
Software and Algorithms		
FlowJo	TreeStar	v9.9.6
Prism	GraphPad Software	v8.4.3
DESeq2	Li and Dewey, 2011	V1.18.1
Ingenuity Pathway Analysis	QIAGEN Inc.	Winter 2019 Release

RESOURCE AVAILABILITY

Lead contact

Further information and requests for resources and reagents should be directed to and will be fulfilled by the Lead Contact (henrik.kloverpris@ahri.org).

Materials availability

This study did not generate new unique reagents.

Data and code availability

The RNA-seq datasets supporting the current study have not been deposited in a public repository because the subjects from which they were generated are at-risk children. The processed expression matrices are available upon request from the lead contact. Access to the raw data will be considered on a case-by-case basis with supporting IRB approval on the behalf of the requestor.

EXPERIMENTAL MODEL AND SUBJECT DETAILS

Peripheral blood (PB) samples from children were obtained from the Ithemabalabantu pediatric cohort in Durban, KwaZulu-Natal (KZN), South Africa (Muenchhoff et al., 2016) and from Stanger Hospital, Stanger, KwaZulu-Natal (KZN), South Africa (Roider et al., 2019). PB samples from newborn/infants were obtained from the Ucwaningo Lwabantwana (meaning learning from children) infant cohort from Edendale, Mahatma Gandhi Memorial, Stanger and Queen Nandi Memorial Hospitals in KZN (Adland et al., 2020). Tonsil tissue samples were obtained from pediatric patients undergoing routine tonsillectomy at Stanger Hospital, Stanger, KwaZulu-Natal (KZN), South Africa (Roider et al., 2019). Informed consent was obtained from all adult study participants; and for underage children and adolescents, informed consent was obtained from their guardians. All HIV infected individuals were infected via vertical transmission from maternal HIV infection. For non-adult participants, 4 age groups were defined: 1. Newborns, aged 3–45 h; 2. Infants, aged 2–60 months; Paediatrics, aged 5–18 years. Paediatric slow progressors (PSP) were defined by stable CD4 T cell percentage of total PBMCs > 20% and found to be clinically healthy, while untreated pediatric progressors (PP) were defined by $CD4^{+}$ T cell percentage of total PBMCs < 20% or otherwise meeting requirements for treatment. The pediatric treated cohort have individuals on antiretroviral therapy (ART). All subjects are from black Sub-Saharan ethnicity. This study was approved by the respective institutional review boards and Biomedical Research Ethics Committee, University of KwaZulu-Natal (UKZN) in Durban, South Africa.

METHOD DETAILS

Cell isolation from human blood and tonsil

Peripheral blood mononuclear cells (PBMCs) were isolated by Histopaque 1077 (Sigma-Aldrich) density gradient centrifugation. Tonsil tissue samples was minced and digested with Collagenase D (0.5 mg/ml; Roche) and DNase I (20 μg/ml; Sigma-Aldrich) for 30 min in 37°C shaking incubator. Digested tissue was passed through 70 μm cell strainers. Lymphocytes from the tonsil were isolated by Histopaque 1077 (Sigma-Aldrich) density gradient centrifugation.

Flow cytometry analysis and cell sorting

For FACS analysis, different antibody panels for phenotype and intracellular cytokine staining (ICS) were used. A complete list of antibodies used with identifier and source information can be found in the Key Resources Table. All samples were surface stained at room temperature for minimum 20 min and near-infrared live/dead cell viability staining kit (Invitrogen). For experiments involving ICS, the cells were stimulated with PMA (5 ng/ml; Sigma) plus Ionomycin (1 μg/ml; Sigma) in the presence of Golgiplug and Golgistop (BD Biosciences) for 4 hr in 37°C incubator. Cells were stained with fluorochrome-conjugated monoclonal antibodies and subsequently fixed, permeabilized, and stained by BD Cytofix/Cytoperm Kit (BD Biosciences). Blocking with 20% goat serum for 20 min was done prior to intracellular antibody staining. After staining cells were washed and fixed in 2% paraformaldehyde before acquisition on a 4 laser, 17 parameter BD FACSAria Fusion flow cytometer within 24 h of staining. Data were analyzed with FlowJo v.9.7.2 (TreeStar). For cell sorting experiments, cells were processed from cryopreserved, surface stained, kept on ice in PBS and sorted immediately after staining. All samples were surface stained at room temperature for a minimum 20 mins. Bulk populations were cell sorted to a purity 99% on the BD FACSAria Fusion flow cytometer.

RNA isolation, library construction, sequencing, and alignment

$CD4^+$ T cells, ILC2s, ILCPs, NK $CD16^{high}$ and NK $CD56^{high}$ cells from PBMCs and $NKp44^+$ ILC3s, $NKp44^-$ ILC3s, NK $CD127^-$ and 4 distinct $CD4^+$ T cell subsets from TMCs (100 cell replicates) were FACS sorted directly into 50 μL of RLT Lysis Buffer (QIAGEN) supplemented with 1% v/v 2-mercaptoethanol. Briefly, 50 μL of mixed lysate from each sample was transferred to a skirted 96 well plate. Genetic material was pulled down and purified by mixing the lysate in each well with 2.2x volumes of Agencourt RNAClean XP SPRI beads (Beckman Coulter) and washing 3x with 75 μL of 80% ethanol. After drying, the SPRI beads were re-suspended in 4 μL of pre-reverse transcription (RT) mix, incubated for 3 min at 72°C, and placed on ice. Next, Smart-Seq2 Whole Transcriptome Amplification (WTA) was performed: 7 μL of RT mix was added to each well and RT was carried out; then, 14 μL of PCR mix was added to each well and PCR was performed. Thereafter a cDNA cleanup was performed using 0.6x and 0.8x volumes of Agencourt AMPure XP SPRI beads (Beckman Coulter) which was then quantified using a Qubit dsDNA HS Assay Kit (Life Technologies). Library size and quality were measured by Bioanalyzer using a High Sensitivity DNA Analysis Kit (Agilent Technologies). Sequencing libraries were prepared from WTA product using Nextera XT (Illumina). After library construction, a final AMPure XP SPRI clean-up (0.8 volumes) was conducted. Library concentration and size were measured with the KAPA Library Quantification kit (KAPA Biosystems) and a TapeStation (Agilent Technologies), respectively. Finally, samples were sequenced on a NextSeq500 (30 bp paired-end reads) to an average depth of 5 million reads. Reads were aligned to hg38 (Gencode v21) using RSEM and TopHat (Li and Dewey, 2011) and estimated counts and transcripts per million (TPM) matrices generated. Any samples with fewer than $5x10^5$ or more than $6x10^6$ aligned reads or fewer than 10,000 uniquely expressed genes were removed from subsequent analysis.

RNA-Seq Differential Expression Analysis

Differential expression analysis was performed using DESeq2 (v1.18.1) (Love et al., 2014). Expected counts from biological replicates for each cell type and participant were averaged prior to differential expression in order to prevent participant specific genes from generating false positives and reduce spurious heterogeneity from small (100-cell) populations. Small populations may show skewed expression based on the cell composition within; thus this replicate averaging approach is particularly important given our limited access to pediatric tissue sources and low frequency of these immune populations in order to remove further bias from small population sorts. See Tables S1 and S6 for replicate numbers. Tonsil analyses for the ILC3 and NK cell subsets were restricted only to samples from female subjects given insufficient age matched male subjects. To calculate DEGs between cell subsets, we used the design ~1 + HIV.Status + Gender + Cell.Type in blood and the design ~1 + HIV.Status + Cell.Type in tonsil. To calculate DEGs between HIV infected children and uninfected controls, we separated the samples by cell subset and used the design ~Gender + HIV.Status for blood subsets and the design ~HIV for tonsil subsets. For the $CD4^+$ T cell subsets in the tonsil, we used the design ~Gender + HIV.Status as sample availability required us to use cells from both female and male participants.

Gene Set Analysis

Gene set analysis was performed using Ingenuity Pathway Analysis (IPA; Winter 2019 Release, QIAGEN Inc.) and Gene Set Enrichment Analysis (GSEA) using the piano package in R (1.18.1). For IPA, DEGs whose FDR corrected $q < 0.1$ were used in the "Core" analysis with the log2FC and q values included in the analysis. To implement GSEA on our DESeq2 results, we used the log2FC of all genes whose FDR corrected $q < 0.1$ as t-value input into the runGSA function with setting the argument geneSetStat = "gsea." We

chose to use the KEGG and GO databases (downloaded from MSigDB v7.0) (Subramanian et al., 2005) for GSEA analysis as these databases are well annotated for metabolic and cellular activation gene sets that are cell-type agnostic.

For the IPA enrichment on tonsil population comparisons, only 1-3 of the significantly enriched terms had non-N/A values for each population. Thus, we have omitted the z-scores from the manuscript.

QUANTIFICATION AND STATISTICAL ANALYSIS

Graphs were plotted using Prism 8.4.3 (GraphPad Inc.) Differences between groups were analyzed using Mann Whitney U-test or Dunn's multiple comparisons test (two-sided) with specific test used stated in the figure legends otherwise. Data are presented as the medians values with boxes representing IQR range and range by error bars, with a p value < 0.05 considered statistically significant. The values of n refers to the number of participants used in study. In the other parts, it refers to the number of dependent experiments.

Cell Reports, Volume *32*

Supplemental Information

Innate Lymphoid Cell Activation and Sustained Depletion in Blood and Tissue of Children Infected with HIV from Birth Despite Antiretroviral Therapy

Alveera Singh, Samuel W. Kazer, Julia Roider, Kami C. Krista, Jane Millar, Osaretin E. Asowata, Abigail Ngoepe, Duran Ramsuran, Rabiah Fardoos, Amanda Ardain, Maximilian Muenchhoff, Warren Kuhn, Farina Karim, Thumbi Ndung'u, Alex K. Shalek, Philip Goulder, Alasdair Leslie, and Henrik N. Kløverpris

Supplementary Material

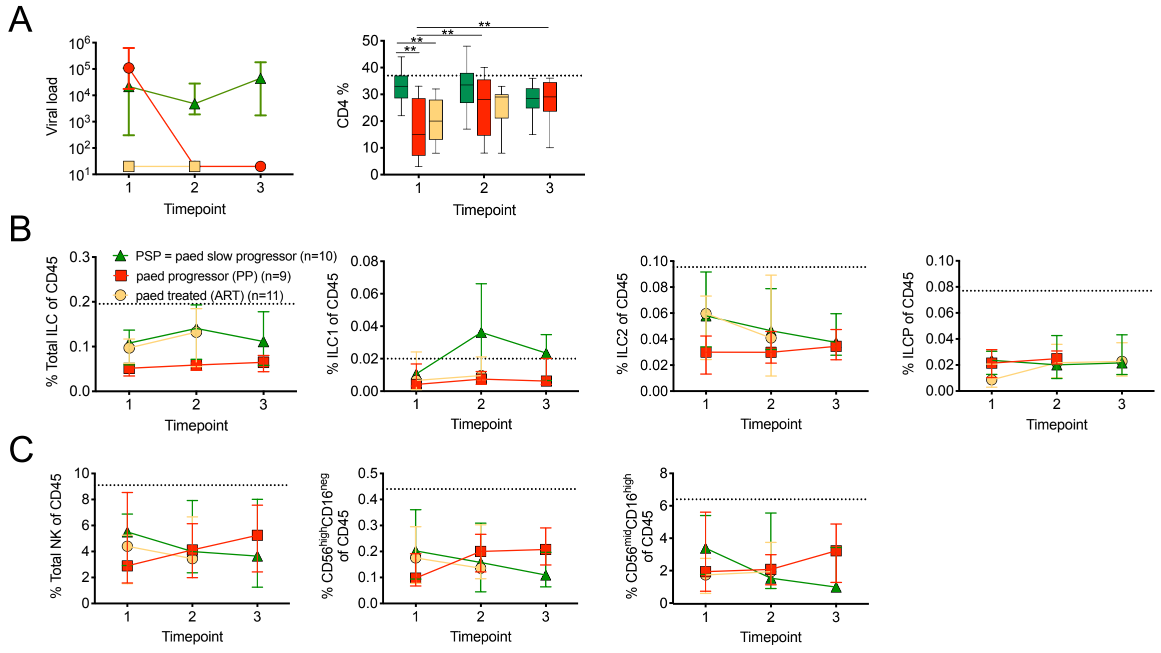

Figure S1. Related to Figure 3. Sustained depletion of all ILC subsets during treated paediatric HIV infection.. (**A**) Plasma viral load (left) and CD4 percentage (right) of 3 paediatric HIV groups: 1. Paediatric slow progressors (PSP, green, n=12), 2. Paediatric progressors (PP, red, n=11) and 3. Paediatric treated (ART, yellow, n=11). (**B**) Same longitudinal sampling as in (A) but showing longitudinal sampling of all blood (total ILC: top row, right; blue), helper (ILC1: middle row, left; yellow; ILC2: middle row, center; red), precursor (ILCP: middle row, right, light blue) and (**C**) cytotoxic subsets (NK cells: bottom row). *P*-values by Kruskall-Wallis multiple comparison.

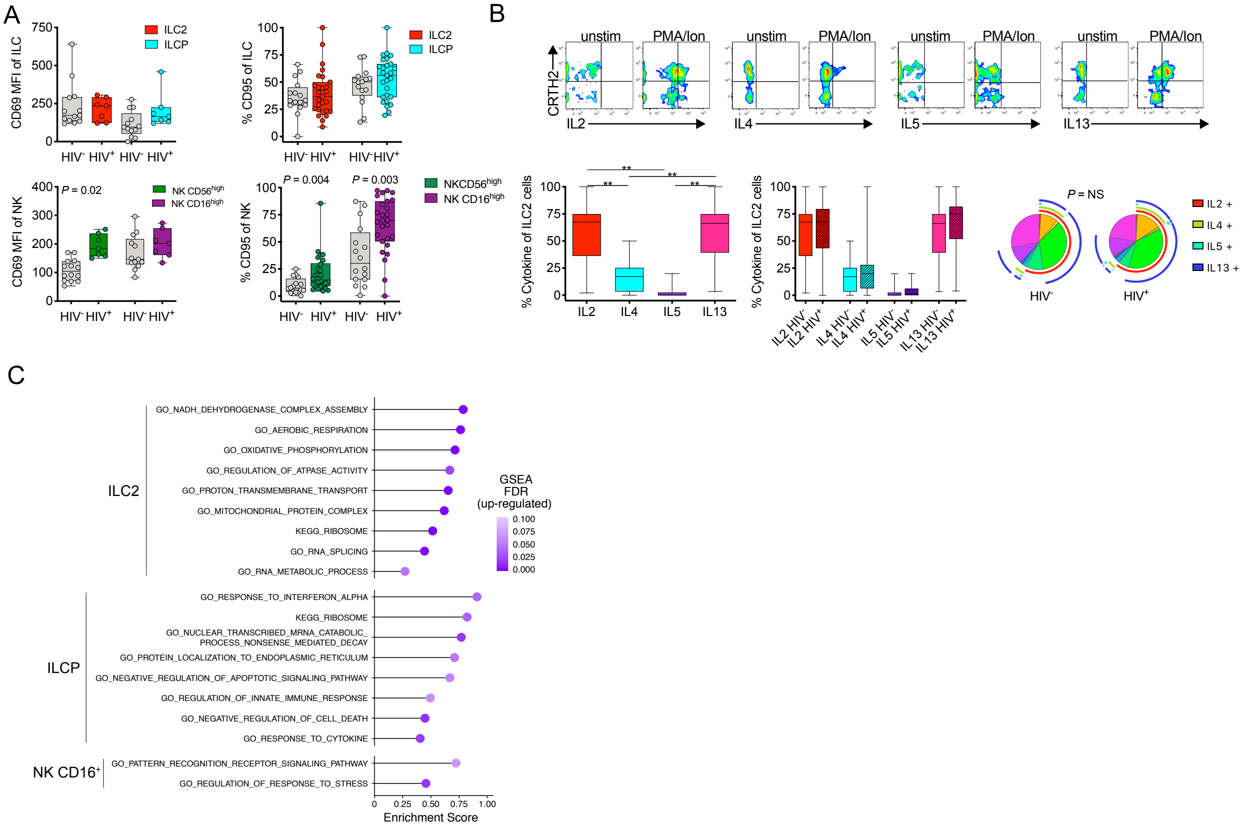

Figure S2. Related to Figure 4. Circulating NK cells, but not helper ILCs, upregulate activation markers in HIV-infected children with ILC2s and ILCPs upregulating genes enriched for metabolic GO and KEGG terms in paediatric HIV infection. (**A**) CD69 median fluorescence intensity (MFI) of CD69 and CD95 gated on ILC2 and ILCP (top) and NK CD56^{high} and NK CD16^{high} subsets (bottom) in blood. (**B**) Intracellular cytokine staining of ILC2 cells after 4 hrs PMA/Ion stimulation. (**C**) GSEA enrichment score and significance plotted of representative hits for DEGs (FDR corrected q<0.1; see Table S3 for genes) for ILC2s, ILC3s, and NK CD16^{+}cells between HIV infected children and healthy controls. See Table S4 for full GSEA results; performed using the GO and KEGG terms available on MSigDB (v7.0).

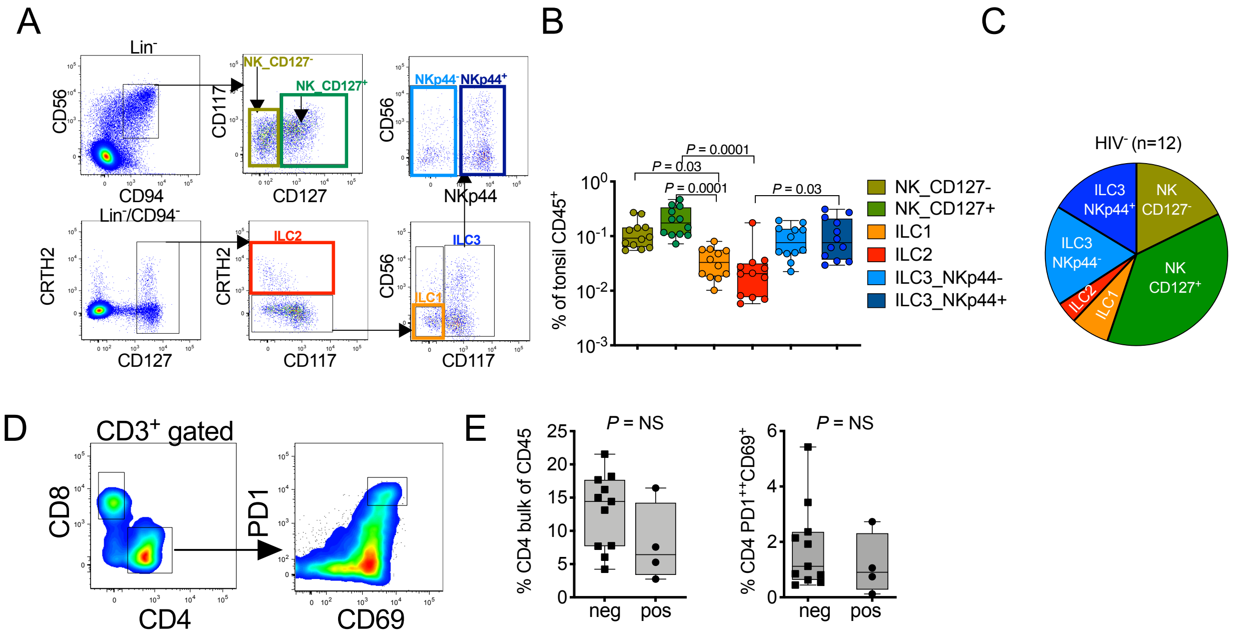

Figure S3. Related to Figure 5. Gating strategy to identify frequencies of helper ILCs and NK cells in paediatric tonsils and with unchanged frequencies of CD4⁺ T cells in HIV infected paediatric tonsils. (**A**) Gating strategy to identify 4 helper and 2 cytotoxic ILC populations in paediatric tonsil lymphocytes from lineage negative (Lin⁻) cells (CD3, CD4, CD19, CD14, TCRαβ, TCRγδ, CD11c). (**B**) Frequencies of each of these 6 innate lymphocyte populations expressed as % of CD45 cells with horizontal bar indicating median values of 12 HIV negative paediatric tonsils. (**C**) Relative frequencies for each of the 6 populations gated as in A. (**D**) Gating strategy to identify frequencies of CD4⁺ bulk T cells and T-follicular helper cells in paediatric tonsils. (**E**) Frequencies expressed as % of CD45 cells with horizontal bar indicating median values of comparison of 12 HIV negative paediatric tonsils against 4 HIV infected paediatric tonsils.

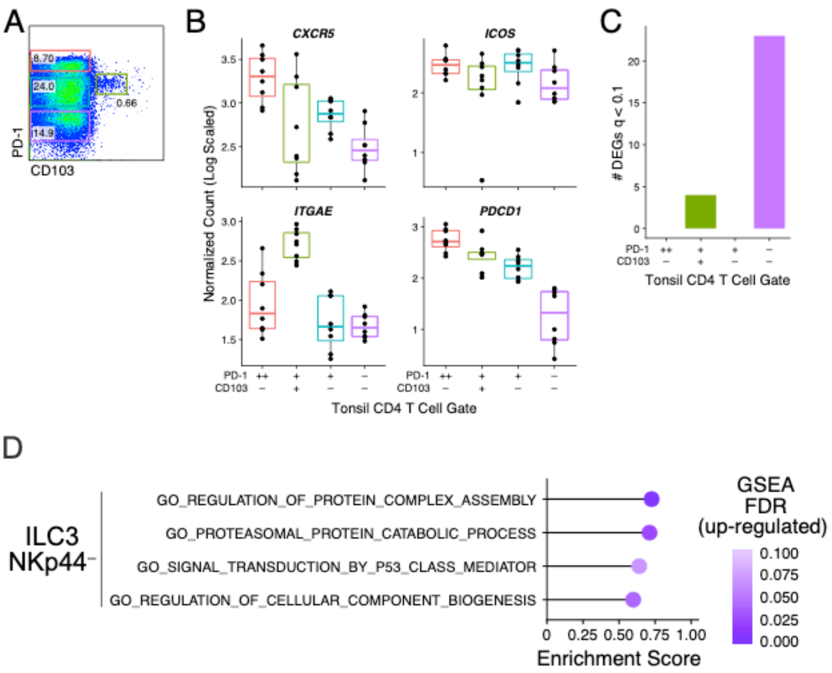

Figure S4. Related to Figure 6. CD4⁺ T cell subsets from tonsils of HIV infected and HIV uninfected children and ILC3 NKp44⁻ cells, but not ILC3 NKp44⁺ or NK CD127⁻ cells, upregulating genes associated with protein production and activation. (**A**) Sort gates used on $CD4^+CD8^-$ cells to isolate (1) PD-1^{++}CD103^- [red]; (2) PD-1^+CD103^+ [green]; (3) PD-1^+CD103^- [blue]; and (4) PD-1^-CD103^- [purple] subsets. (**B**) Gene expression of *CXCR5, ICOS, ITGAE* (CD103), and *PDCD1* (PD-1) across subsets and participants. Each dot represents the average of 1-3 biological replicates of the subset for a given participant. (**C**) Number of differentially expressed genes for each tonsil $CD4^+$ T cell subset between HIV infected (n=3; 1 male + 2 female) and HIV uninfected (n=5; 4 male + 1 female) children. FDR q calculated using DESeq2. **(D)** GSEA enrichment score and significance plotted of representative hits for DEGs (FDR corrected q<0.1; see Table S7 for genes) for ILC3 NKp44⁻ cells between HIV infected children and healthy controls. See Table S8 for full GSEA results; performed using the GO and KEGG terms available on MSigDB (v7.0).
